# Bat parasites (Acari, Anoplura, Cestoda, Diptera, Hemiptera, Nematoda, Siphonaptera, Trematoda) in France (1762–2018): a literature review and contribution to a checklist

**DOI:** 10.1051/parasite/2020051

**Published:** 2020-11-17

**Authors:** Clément Léger

**Affiliations:** 1 Muséum national d’Histoire naturelle, Direction Générale Déléguée aux Collections (DGD-C) – Service Recherche, Enseignement, Expertise CP 20 38 rue Geoffroy Saint-Hilaire 75005 Paris France

**Keywords:** Bibliometry, Chiroptera, Host-parasite associations, Parasite biodiversity

## Abstract

This paper is a bibliographical survey of records of bat parasites in France (including Corsica) between 1762 and 2018. In total, 237 scientific publications were analysed. They show that bats are infected with a large diversity of endoparasites and ectoparasites. A total of 113 parasite taxa were identified from 27 host species; in addition, six bats were not identified to the species-level. The helminth fauna of bats comprises three species of Cestoda, 15 of Trematoda, and 13 of Nematoda. Acari parasites include 53 species (in addition to 22 invalid species). Finally, insect parasites comprise 13 species of Diptera (bat flies), 12 of Siphonaptera (fleas), 3 of Hemiptera (bugs), and 1 Anoplura species. Bat taxa reported with parasites were *Barbastella barbastellus, Eptesicus serotinus, Hypsugo savii, Miniopterus schreibersii, Myotis bechsteinii, M. blythii, M. capaccinii, M. dasycneme, M. daubentonii, M. emarginatus, M. myotis, M. mystacinus, M. nattereri, M. punicus, Nyctalus lasiopterus, N. leisleri, N. noctula, Pipistrellus kuhlii, P. nathusii, P. pipistrellus, Plecotus auritus, P. austriacus, Rhinolophus euryale, R. ferrumequinum, R. hipposideros, R. mehelyi, Tadarida teniotis, Eptesicus* sp., *Myotis* sp., *Pipistrellus* sp., *Plecotus* sp., *Rhinolophus* sp. and the species complex *Pipistrellus pipistrellus/kuhlii/nathusii*. As regards *E. nilssonii, Vespertilio murinus* (Particoloured Bat)*, M. alcathoe, M. escalerai, P. macrobullaris* and *P. pygmaeus,* no records were found. These published field data originated from 72 of the 96 departments in metropolitan France. The most commonly cited were Ardèche, Ariège, Bouches-du-Rhône, Haute-Savoie, Maine-et-Loire, Moselle, Meurthe-et-Moselle, Pyrénées-Orientales, Sarthe, Haute-Corse and Corse-du-Sud.

## Abstract (fr)

Cet article est une étude bibliographique des signalements de parasites de chauves-souris en France (y compris la Corse) entre 1762 et 2018. Au total, 237 publications scientifiques ont été analysées. Elles montrent que les chauves-souris sont infectées par une grande diversité d’endoparasites et d’ectoparasites. Un total de 113 taxons parasites ont été identifiés parmi 27 espèces hôtes (en outre, six chauves-souris n’ont pas été identifiées au niveau de l’espèce). La faune helminthique des chauves-souris comprend trois espèces de Cestodes, 15 de Trématodes et 13 de Nématodes. Les Acariens parasites comprennent 53 espèces (en plus de 22 espèces invalides). Enfin, les insectes parasites comprennent 13 espèces de Diptères, 12 de Siphonaptères (puces), 3 d’Hémiptères (punaises) et 1 espèce d’Anoploure. Les taxons de chauves-souris signalés avec des parasites étaient *Barbastella barbastellus, Eptesicus serotinus, Hypsugo savii, Miniopterus schreibersii, Myotis bechsteinii, M. blythii, M. capaccinii, M. dasycneme, M. daubentonii, M. emarginatus, M. myotis, M. mystacinus, M. nattereri, M. punicus, Nyctalus lasiopterus, N. leisleri, N. noctula, Pipistrellus kuhlii, P. nathusii, P. pipistrellus, Plecotus auritus, P. austriacus, Rhinolophus euryale, R. ferrumequinum, R. hipposideros, R. mehelyi, Tadarida teniotis, Eptesicus* sp., *Myotis* sp., *Pipistrellus* sp., *Plecotus* sp., *Rhinolophus* sp. et le complexe d’espèces cryptiques *Pipistrellus pipistrellus/kuhlii/nathusii*. Aucun signalement n’a été trouvé pour *E. nilssonii, Vespertilio murinus, M. alcathoe, M. escalerai, P. macrobullaris* et *P. pygmaeus*. Ces données de terrain publiées proviennent de 72 des 96 départements de France métropolitaine. Les plus cités sont l’Ardèche, l’Ariège, les Bouches-du-Rhône, la Haute-Savoie, le Maine-et-Loire, la Moselle, la Meurthe-et-Moselle, les Pyrénées-Orientales, la Sarthe, la Haute-Corse et la Corse-du-Sud.

## Introduction

Bats (Mammalia: Chiroptera) represent the second-most diverse order of mammals, after rodents. As of 2007, 42 bat species have been reported from Europe (Dietz *et al*. [84]). According to Arthur & Lemaire [16], 35 species have been unambiguously identified in France. Many aspects of the ecology of bats are under study (e.g. swarming, hunting sites, flight routes, habitat studies, acoustic ecology). One of these aspects is the study of bat parasites, which has a long history in Europe, for instance in French-speaking areas (France, Belgium). Bats are infected with a large diversity of parasites. Around the year 1999, *c.* 756 taxa were known to be associated with bats worldwide [167]. In Lanza’s book, a wide range of parasitic organisms were presented, belonging to 13 groups: Myconta (two taxa), Acanthocephala (three taxa), Mallophaga (one taxon, accidental exposure), Anoplura (two taxa), Heteroptera (11 taxa), Neobacteria (*c.* 31 taxa), Protozoa (25 taxa), Cestoda (55 taxa), Digenea (105 taxa), Nematoda (62 taxa), Acari (324 taxa), Diptera (65 taxa) and Siphonaptera (64 taxa). This includes at least ten phyla: Acanthocephala (Spiny-headed worms), Apicomplexa, Arthropoda, Ascomycota (Ascomycete fungi), Euglenozoa, Firmicutes, Nematoda, Platyhelminthes, Protobacteria and Spirochaetes. Similar findings were noted by Stiles & Nolan [235] in their “key catalogue” of bat parasites. In addition to the high diversity of bat parasites, these findings point out the predominant share, in the published records, of metazoan parasites. They also point out the issue of diseases in bats and the issue of bat parasites as disease vectors for their hosts. Indeed, we know that bats are hosts to a large range of infections (transmission linked with their ecology) and they seemingly are able to control these infections so that they are mostly asymptomatic. Some bat parasites (e.g. bat flies) are known to be disease vectors for their hosts [83, 129, 181, 184, 192, 261].

**Figure 1:**
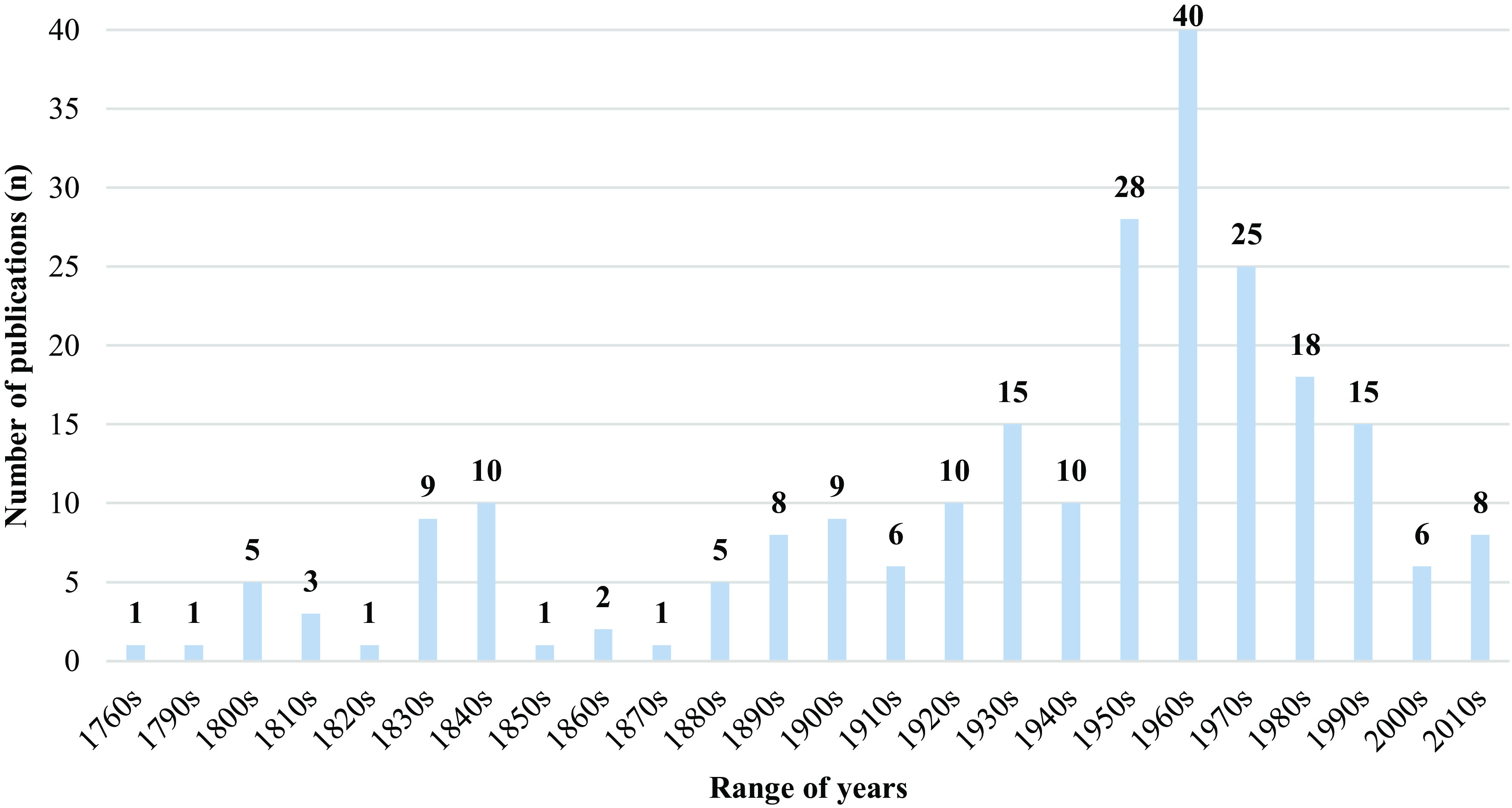
Number of studies (*n* = 237) that include bat parasites observed in France since 1760, by decade.

Among the earliest works on bat parasites in France is Étienne-Louis Geoffroy’s *Histoire abrégée des insectes* [123], published in 1762 (Fig. 1). This book marks the starting point for research on bat parasites in France. The present paper reviews metazoan parasites reported on bats in France between 1762 and 2018, with the exception of acanthocephalans. According to the Host-Parasite Database of the Natural History Museum, London [125], no bat parasites belonging to the Acanthocephala phylum are currently known in France. In addition, hyperparasites are excluded from this paper. Nevertheless, it should be noted that bat parasites have their own parasites, such as Laboulbeniales fungi associated with bat flies or viruses of haemosporidian parasites. Some of these hyperparasites have reports from France, specifically in the department of Gard (*Arthrorhynchus eucampsipodae* Thaxt., 1901 and *A. nycteribiae* (Peyr.) Thaxt., 1931) [55, 129, 238]. The purpose of the present paper is twofold: the primary aim is to summarize the large body of published field data; and secondly to inform the reader about the geographical origin of the data and to contribute to a general overview and checklist of bat-parasite associations in France.

## Methods

Initially, I used the works of nine authors: Anciaux de Faveaux [5–7], Beaucournu [28, 29], Beaucournu and Launay [37], Hůrka [149], Lanza [167], Maa [185] and Szentiványi *et al.* [237]. The list of all the sources used in these papers offers an essential bibliographical guide. The online catalog of the Library of the Muséum National d’Histoire Naturelle (MNHN) was also used. I checked all available publications on each of the searched terms including a combination of France or the name of administrative departments (*n* = 111) or the names of former administrative regions of France (*n* = 22) with one of the generic names of the bat parasites, as mentioned in *I parassiti dei pipistrelli (Mammalia, Chiroptera) della fauna italiana* [167], *Parasite diversity of European* Myotis *species with special emphasis on* Myotis myotis *(Microchiroptera, Vespertilionidae) from a typical nursery roost* [121], *Les puces de France et du basin méditerranéen occidental* [37], and *Checklist of host associations of European bat flies (Diptera: Nycteribiidae, Streblidae)* [237]. For the study area, see Figure 5. I searched Google Scholar, ISI Web of Science, Hyper-Article en Ligne (HAL), Biodiversity Heritage Library (BHL), Gallica, and Archives. The collated sources (*n* = 237) were then analysed. I then proceeded to index them in terms of their chronology, taxonomy, and geography. The validity of all the taxa found was checked using the comprehensive synonymies provided by the Host-Parasite Database of the Natural History Museum, London [125] and the Inventaire National du Patrimoine Naturel of the Muséum National d’Histoire Naturelle (MNHN) [153]. The taxonomic works of Fain [106–108], Lanza [167], Neumann [197], Da Fonseca [72], Radovsky [212], Roy and Chauve [221, 222], Rudnick [223], Stiles and Nolan [235] and Theodor & Moscona [241] were also used. The bat classification and taxonomy, in this paper, is based on Dietz *et al.* (2009) and Arthur and Lemaire (2015). Authorities for the host taxa and parasites species are given in Tables 1 and 2. The map in Figure 5 was created using Carto-SI (https://www.carto-si.com/).

## Results and discussion

**Figure 2:**
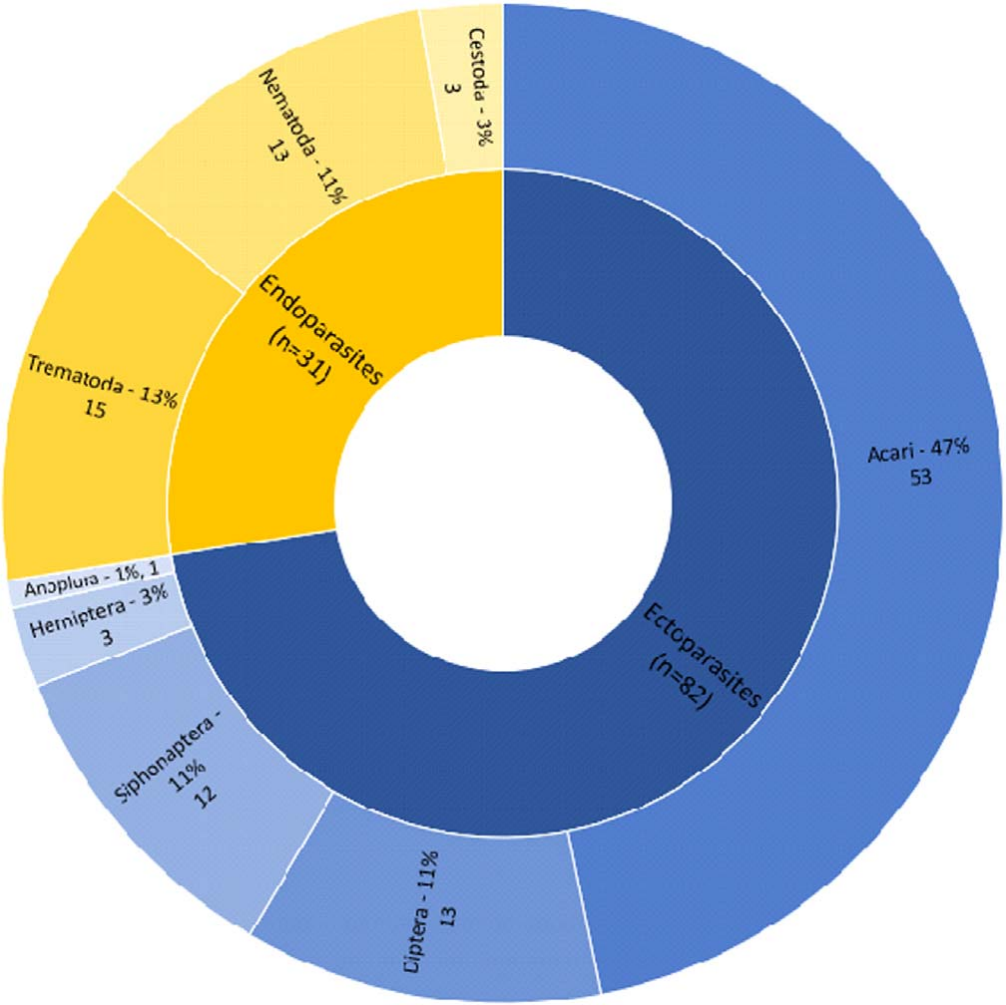
Overview of the 113 generally recognised parasite taxa that are mentioned in the analysed papers (*n* = 237) per host taxonomic group. Invalid species (*n* = 22 Acari and 3 Diptera) recorded in the literature, records reported from France without identification to species level (*n* = 6 Acari; 1 Cestoda; 2 Diptera; 1 Hemiptera; 2 Nematoda and 2 Trematoda) and species only noted as absent (*n* = 3 Acari and 1 Diptera) are not included here.

Based on published data, eight groups of bat parasites reported from France have been identified (Fig. 2). The majority of the analysed papers (94%) were published between 1762 and 1999 (Fig. 1). All host-parasite associations are listed in Tables 1 and 2. What follows is an overview of all bat parasites, arranged by higher taxonomic group.

### 1 Phylum Arthropoda Latreille, 1829

#### 1.1 Subphylum Chelicerata Heymons, 1901

##### 1.1.1 Subclass Acari Leach, 1817

**Figure 3:**
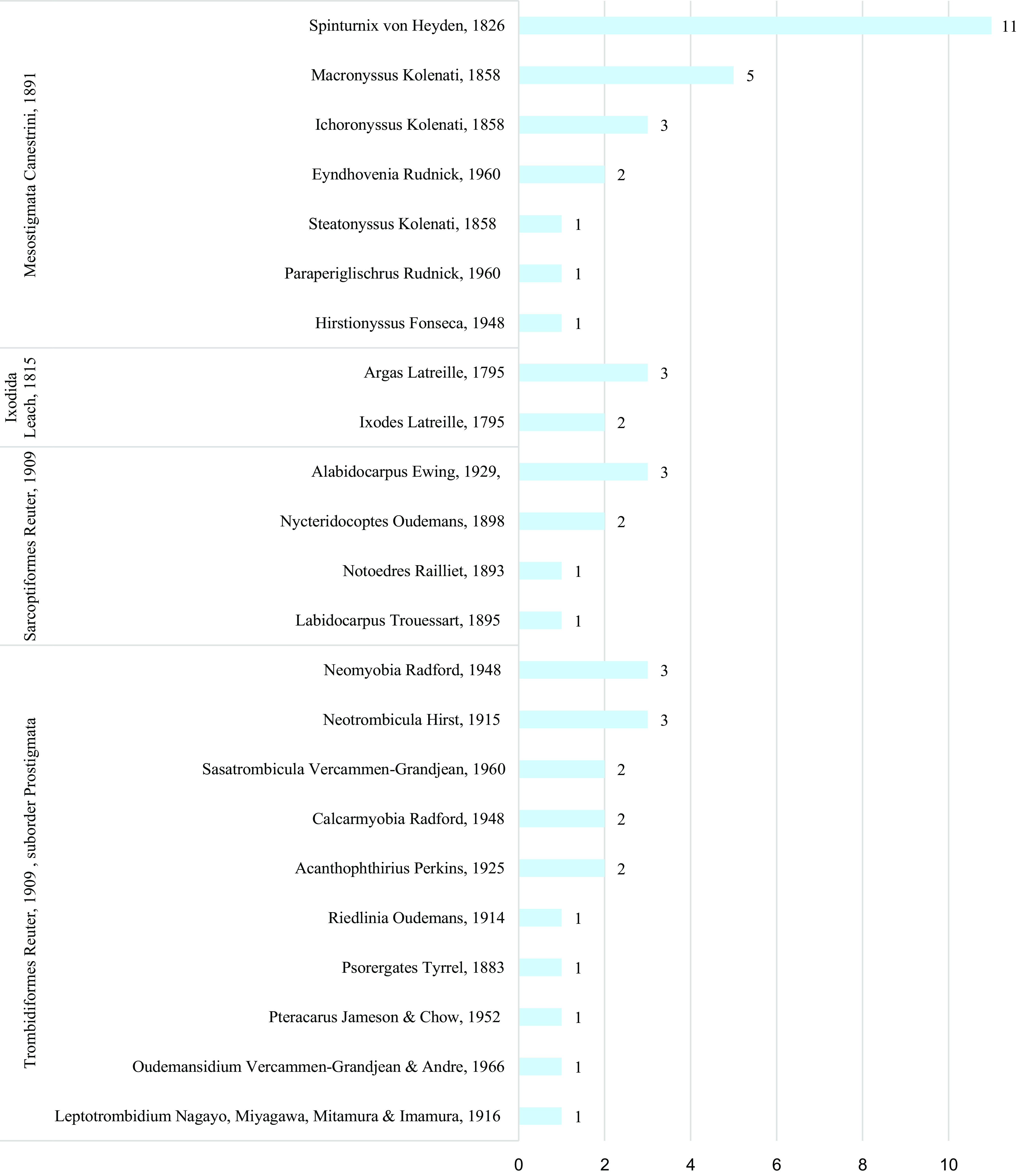
Number of recognised species of Acari (*n* = 53), per host order (*n* = 4) and genus (*n* = 23). Invalid species (*n* = 22 Acari) recorded in the literature (*n* = 237 papers) and species only noted as absent (*n* = 3 Acari) are not included here.

Most of the studied papers (*n* = 112) deal with 75 species (53 generally recognised species and 22 invalid species) of Acari of four groups: Ixodida, Mesostigmata, Trombidiformes (suborder Prostigmata), and Sarcoptiformes (Fig. 3). A total of 53 recognised species (including two subspecies) of mites and ticks were reported to be parasites of bats in France prior to 2018. Of these, two species have only been collected from the border between Switzerland and the French department of Haute-Savoie (Col de Bretolet): *Spinturnix helvetiae* and *S. acuminatus.*

These recognised species found in the literature (*n* = 53) reported from France belong to 23 genera: *Acanthophthirius*, *Alabidocarpus*, *Argas, Calcarmyobia, Eyndhovenia*, *Hirstionyssus*, *Ichoronyssus*, *Ixodes*, *Labidocarpus, Leptotrombidium*, *Macronyssus, Neomyobia, Neotrombicula, Notoedres, Nycteridocoptes, Oudemansidium*, *Paraperiglischrus, Pteracarus*, *Psorergates, Riedlinia, Sasatrombicula, Steatonyssus* and *Spinturnix*. Among this group, the most diverse genera reported in France are *Spinturnix* and *Macronyssus* (Fig. 3): these genera account for 30% of all documented Acari infections involving valid species.

Six more records reported from France without identification to species level were found. These comprise *Dermanyssus* sp. [86], *Ichoronyssus* sp. [25], *Ixodes* sp. [178], *Neomyobia* sp. [126], *Steatonyssus* sp. [152, 212] and *Spinturnix* sp. [133]. Three other species are noted as absent from bats in western France, namely *Ixodes canisuga*, *I. ricinus*, and *Pholeoixodes hexagonus* [28]. Finally, 22 invalid taxa reported from France were found in the analysed papers. Examples for this category are *Dermanyssus murinus* (Lucas, 1840) [101, 104, 167, 183, 195]*, D. vespertilionis* Dugès, 1834 [94]*, Pteroptus vespertilionis*, and *Spinturnix vespertilionis* (C.L. Koch)] [70].

**Table 1:** List of bat species and their associated metazoan parasites in France (including Corsica), based on the published literature. Authors are listed in the bibliography. See also the work titled *Les parasites métazoaires des Chiroptères de France (Acari, Anoplura, Cestoda, Diptera, Hemiptera, Nematoda, Siphonaptera, Trematoda) : contribution à un état des lieux bibliographique (1762–2018) et à l’établissement d’une liste nationale* (2019). Invalid species are listed in brackets. Records marked with an exclamation mark (!) are invalid. Records marked with a question mark (?) are dubious. They may require further clarification.

Bat host	Number of parasite species reported	Parasite occurrence and citation
*Barbastella barbastellus* (Schreber, 1774)	10 species (9 recognised species and 1 invalid species)	**Acari**
		Absence of *Ixodes canisuga* Johnston, 1849 [28]
		Absence of *Pholeoixodes hexagonus* (Leach, 1815) [28]
		[*Pteroptus vespertilionis* L. Duf. [=Dufour] = *Pteroptus vespertilionis* (Dufour, 1832] [127, 177]
		[*Pteroptus vespertilionis*] [67]
		Acanthophthirius (Myotimyobia) pantopus (Poppe et Trouessart, 1895) [102, 114, 167, 235, 249]
		*Argas vespertilionis* (Latreille, 1796) [28]
		*Ixodes (Eschatocephalus) vespertilionis* C.L. Koch, 1844 [28, 32, 200]
		*Spinturnix punctata* (Sundevall, 1833) [80, 167]
		See also [128]
		**Siphonaptera**
		*Ischnopsyllus (Hexactenopsylla) hexactenus* (Kolenati, 1856) [2, 29, 33, 37, 38, 44, 200]
		*Ischnopsyllus (Ischnopsyllus) octactenus* (Kolenati, 1856) [200, 228]
		*Ischnopsyllus (Ischnopsyllus) simplex* Rothschild, 1906 [29, 37]
		*Nycteridopsylla longiceps* Rothschild, 1908 [29, 33, 37, 167]
		*Nycteridopsylla pentactena* (Kolenati, 1856) [2, 29, 33, 37, 44, 167, 200]
Chiroptera gen. sp.	30 species	**Acari**
	(22 recognised species and 8 invalid species)	[*Acarus vespertilionis* Hermann] [257, 258]
	1 innominate species	[*Dermanyssus vespertilionis* Dugès, 1834] [94]
		[*Leiognathus armatus* (=*Hirstionyssus arcuatus* (C.L. Koch, 1839)?)] [160, 177]
		[*Liponyssus setosus* (Kolenati)] [72]
		[*Nycteribia vespertilionis* Meig.] [12]
		[*Pteroptus vespertilionis* Dufour] [120, 177]
		[*Pteroptus vespertilionis*] [160]
		*Argas (Secretargas) transgariepinus* White, 1846 [189]
		*Ixodes (Eschatocephalus) vespertilionis* C.L. Koch, 1844 [45, 69, 160, 190, 197–199, 248]
		*Macronyssus uncinatus* (Canestrini, 1885) [160, 177]
		*Neotrombicula autumnalis* (Shaw, 1790) [62]
		*Sasatrombicula* (*Sasatrombicula*) *hexasternalae* (Vercammen-Grandjean, 1963) [7]
		Spinturnix myoti (Kolenati, 1856) [7, 65?, 167, 223, 246]
		*Spinturnix plecotina* (C.L. Koch, 1839) [7]
		*Spinturnix psi* (Kolenati, 1856) [7, 167, 210, 223]
		**Diptera**
		[*Nycteribia vespertilionis* Meig.] [12]
		*Nycteribia (Nycteribia) pedicularia* Latreille, 1805 ?[170]
		*Phthiridium biarticulatum* Hermann, 1804 ?[173, 204]
		*Nycteribia (Nycteribia) latreillii* (Leach, 1817) [148]
		*Nycteribia (Nycteribia) schmidlii* Schiner, 1853
		*Penicillidia (Penicillidia) dufourii* (Westwood, 1834) [43]
		**Hemiptera**
		*Cimex dissimilis* (Horváth, 1910) [207]
		*Cimex lectularius* Linnaeus, 1758 [28, 35]
		*Cimex pipistrelli* Jenins, 1839 [28, 236]
		*Cimex* sp. [28, 177]
		**Nematoda**
		*Molinostrongylus alatus* (Ortlepp, 1932)? [167]
		**Siphonaptera**
		*Ischnopsyllus (Hexactenopsylla) hexactenus* (Kolenati, 1856) [142]
		*Ischnopsyllus (Ischnopsyllus) intermedius* (Rothschild, 1898) [44]
		*Ischnopsyllus (Ischnopsyllus) octactenus* (Kolenati, 1856) [219]
		*Nycteridopsylla dictena* (Kolenati, 1856) ! see Beaucournu et Launay (1990; p. 314 [paper n°36]) [37, 146, 167, 227]
		*Nycteridopsylla pentactena* (Kolenati, 1856) [204]
		
*Eptesicus serotinus* (Schreber, 1774)	17 species	**Acari**
	(16 recognised species and 1 invalid species)	Absence of *Ixodes canisuga* Johnston, 1849 [28]
	1 innominate species	Absence of *Pholeoixodes hexagonus* (Leach, 1815) [28]
		[*Pteroptus vespertilionis* (Dufour, 1832)] [190]
		*Argas (Carios) vespertilionis* (Latreille, 1802) [31, 45, 86]
		*Argas (Secretargas) transgariepinus* White, 1846 [45, 189]
		*Argas vespertilionis* (Latreille, 1796) [28, 38]
		*Ixodes (Eschatocephalus) vespertilionis* C.L. Koch, 1844 [28, 32, 166]
		*Notoedres (Notoedres) chiropteralis* (Trouessart, 1896) [48, 106, 167, 250]
		*Spinturnix psi* (Kolenati, 1856) [48]
		**Cestoda**
		*Vampirolepis acuta* (Rudolphi, 1819) [158, 159, 177, 196, 255]
		*Vampirolepis balsaci* (Joyeux & Baer, 1934) [158, 159, 167, 177, 180, 196, 255]
		*Vampirolepis sp./Milina sp.* [86]
		**Diptera**
		*Basilia (Basilia) mediterranea* Hůrka, 1970 [43, 167, 237]
		*Basilia (Basilia) nattereri* (Kolenati, 1857) [241]
		*Nycteribia (Nycteribia) kolenatii* Theodor & Moscona, 1954 [147, 241]
		**Siphonaptera**
		*Ischnopsyllus (Ischnopsyllus) intermedius* (Rothschild, 1898) [29, 33, 37, 38, 142, 167, 227]
		*Nycteridopsylla pentactena* (Kolenati, 1856) [29, 33, 37, 164, 167]
		**Trematoda**
		*Lecithodendrium linstowi* Dollfus, 1931 [68, 88, 167]
		*Plagiorchis vespertilionis* (O.F. Müller, 1784) [68, 167]
		*Prosthodendrium (Prosthodendrium) longiforme* (Bhalerao, 1926) [88, 167]
*Eptesicus* sp.	1 recognised species	**Acari**
		*Neotrombicula racovitzai* Feider, 1970 [6, 118, 167]
*Hypsugo savii* (Bonaparte, 1837)	4 recognised species	**Acari**
		*Argas (Carios) vespertilionis* (Latreille, 1802) [45]
		*Argas (Secretargas) transgariepinus* White, 1846 [45, 189]
		*Spinturnix nobleti* Deunff, Volleth, Keller et Aellen, 1990 [81, 167]
		**Diptera**
		*Basilia (Basilia) mediterranea* Hůrka, 1970 [41, 167, 237]
*Miniopterus schreibersii* (Natterer *in* Kuhl, 1817)	38 species	**Acari**
	(33 recognised species and 5 invalid species)	[Pteroptus vespertilionis (Dufour, 1832)] [203]
		[*Spinturnix vespertilionis*] [24]
		[*Spinturnix vespertilionis* (C.L. Koch)] [70]
		*Argas vespertilionis* (Latreille, 1796) ? [85]
		*Calcarmyobia rhinolophia* (Radford, 1940)? There is only one case. According to Lanza (1999), this field data published by Beron (1971) is doubtful: it could be *Calcarmyobia dusbabeki* Uchikawa, 1985 [50, 51, 167]
		*Eyndhovenia euryalis* (G. Canestrini, 1885) [76]
		*Ichoronyssus diversipilis* Vitzthum, 1920 [24]
		*Ichoronyssus spinosus* (Oudemans) 1902) [24, 70, 72]
		Ixodes (Eschatocephalus) simplex simplex Neumann, 1906 [15, 31, 32, 45, 51, 143, 166, 167, 176] see Beaucournu (1966; p. 498) paper n°30
		*Ixodes (Eschatocephalus) vespertilionis* C.L. Koch, 1844 [6, 28, 176]! see Beaucournu (1966; p. 498) paper n°30 [200, 242]
		*Macronyssus granulosus* (Kolenati, 1856) [50, 167]
		Spinturnix myoti (Kolenati, 1856) [76]
		*Spinturnix psi* (Kolenati, 1856) [50, 63, 76, 139]
		**Cestoda**
		*Milina grisea* van Beneden, 1873 [68, 86, 167, 200?]
		**Diptera**
		[*Nycteribia vespertilionis* Meig.] [203]
		*Nycteribia (Nycteribia) pedicularia* Latreille, 1805 ! [85]
		*Phthiridium biarticulatum* Hermann, 1804 ? [85, 126, 237]
		*Nycteribia (Nycteribia) kolenatii* Theodor & Moscona, 1954 [200]
		*Nycteribia (Nycteribia) latreillii* (Leach, 1817) [1, 167]
		*Nycteribia (Nycteribia) schmidlii* Schiner, 1853 [1, 24, 28, 30, 43, 115, 149, 154, 167, 242]; see also [117, 150]
		*Nycteribia (Achrocholidia) vexata* Westwood, 1835 [1, 43, 85, 167]
		*Penicillidia (Penicillidia) dufourii* (Westwood, 1834) [1, 24, 30, 237]
		*Penicillidia (Neopenicillidia) conspicua* Speiser, 1901 ([1, 28, 30, 115, 154, 167, 242], see also [117, 150, 237])
		**Nematoda**
		*Litomosa ottavianii* Lagrange & Bettini, 1948 [19, 167]
		*Molinostrongylus panousei* Dollfus, 1954 [99, 167]
		*Molinostrongylus tipula* (Beneden, 1873) [200]
		*Riouxgolvania rhinolophi* (Bain & Chabaud, 1968) [21, 167]
		*Strongylacantha glycirrhyza* van Beneden, 1873 [99, 167, 200]
		*Trichosomum speciosum* van Beneden, 1873 [200]
		See also [174]
		**Siphonaptera**
		*Nycteridopsylla pentactena* (Kolenati, 1856) [37, 164, 167]
		**Trematoda**
		[*Paralecithodendrium chilostomum* (Mehlis) (=*Prosthodendrium (Prosthodendrium) chilostomum* (Mehlis, 1831) ?)] [200]
		*Lecithodendrium granulosum* Looss, 1907 ? [200]
		*Lecithodendrium linstowi* Dollfus, 1931 [68, 167]
		*Mesotretes peregrinus* (Braun, 1900) [68, 167, 188]
		*Parabascus lepidotus* Looss, 1907 [68, 167]
		*Plagiorchis vespertilionis* (O.F. Müller, 1784) [68, 167, 200, 235]
		*Prosthodendrium (Prosthodendrium) chilostomum* (Mehlis, 1831) [68, 167, 200, 235]
		*Prosthodendrium parvouterus* (Bhalerao, 1926) [68, 167]
		See also [174]
		
*Myotis bechsteinii* (Leisler *in* Kuhl, 1817)	4 recognised species	**Acari**
		Absence of *Ixodes canisuga* Johnston, 1849 [28]
		Absence of *Pholeoixodes hexagonus* (Leach, 1815) [28]
		*Ixodes (Eschatocephalus) vespertilionis* C.L. Koch, 1844 [28, 32, 38]
		*Spinturnix bechsteini* (Deunff, Walter, Bellido et Volleth, 2004) [82, 130]
		**Cestoda**
		*Vampirolepis balsaci* (Joyeux & Baer, 1934) [159, 167, 177, 180, 196, 255]
		**Diptera**
		*Basilia (Basilia) nana* Theodor & Moscona, 1954 [4, 28, 30, 38, 237]
*Myotis blythii* (Tomes, 1857) and *M. blythii oxygnathus* Monticelli, 1885	9 recognised species	**Acari**
	1 innominate species	Absence of *Ixodes (Eschatocephalus) simplex simplex* Neumann, 1906 [32]
		*Nycteridocoptes poppei* Oudemans, 1898 [113]
		*Spinturnix myoti* (Kolenati, 1856) [14, 76]
		**Diptera**
		*Nycteribia (Achrocholidia) vexata* Westwood, 1835 [30]
		*Nycteribia (Nycteribia) latreillii* (Leach, 1817) [30, 167]
		*Nycteribia (Nycteribia) pedicularia* Latreille, 1805 [115, 154, 167]
		*Nycteribia (Nycteribia) schmidlii* Schiner, 1853 [30]
		*Penicillidia (Penicillidia) dufourii* (Westwood, 1834) [14, 43, 115, 154, 237]
		**Nematoda**
		*Riouxgolvania nyctali* (Bain & Chabaud, 1979) [21, 167]
		*Riouxgolvania* sp. [86]
		**Siphonaptera**
		*Ischnopsyllus (Hexactenopsylla) hexactenus* (Kolenati, 1856) ? [14]
*Myotis capaccinii* (Bonaparte, 1837)	9 recognised species	**Acari**
		Spinturnix myoti (Kolenati, 1856) [73]
		*Spinturnix psi* (Kolenati, 1856) [63, 76, 121, 139]
		**Diptera**
		*Nycteribia (Nycteribia) pedicularia* Latreille, 1805 [1, 30, 85]
		*Penicillidia (Penicillidia) dufourii* (Westwood, 1834) [1, 237]
		**Siphonaptera**
		*Nycteridopsylla longiceps* Rothschild, 1908 [37, 164, 167]
		*Nycteridopsylla pentactena* (Kolenati, 1856) [37, 164, 167]
		**Trematoda**
*-*		*Lecithodendrium granulosum* Looss, 1907 [68]
		*Lecithodendrium linstowi* Dollfus, 1931 [68, 167]
		*Plagiorchis vespertilionis* (O.F. Müller, 1784) [68, 167]
*Myotis dasycneme* (Boie, 1825)	1 recognised species	**Acari**
		*Argas (Carios) vespertilionis* (Latreille, 1802) [6, 31, 85]
*Myotis daubentonii* (Leisler *in* Kuhl, 1817) and *Myotis daubentonii nathalinae*	19 species (17 recognised species and 2 invalid species)	**Acari**
	1 innominate species	Absence of *Ixodes canisuga* Johnston, 1849 [28]
		Absence of *Pholeoixodes hexagonus* (Leach, 1815) [28]
		[*Pteroptus trouessarti* Mégnin (=*Haemomyson trouessarti* (Mégnin)?)] [248]
		*Ixodes (Eschatocephalus) vespertilionis* C.L. Koch, 1844 [28, 32]
		*Leptotrombidium russicum* (Oudemans, 1902) [122, 167, 177, 256] see also [10]
		*Neotrombicula autumnalis* (Shaw, 1790) [8–10, 122, 167, 177, 256]
		Nycteridocoptes poppei Oudemans, 1898 [113]
		*Oudemansidium musca* (Oudemans, 1906) [122, 167, 177]
		*Spinturnix andegavinus* (Deunff, 1977) [76]
		**Diptera**
		[*Nycteribia eparticulata*] [248]
		*Nycteribia (Nycteribia) pedicularia* Latreille, 1805 ! [85]
		*Basilia (Basilia) nattereri* (Kolenati, 1857) [187]
		*Nycteribia (Nycteribia) kolenatii* Theodor & Moscona, 1954 [1, 28, 38, 43, 167, 241]
		*Nycteribia (Nycteribia) schmidlii* Schiner, 1853 [147]
		*Nycteribia* sp. [247]
		**Siphonaptera**
		*Ischnopsyllus (Ischnopsyllus) simplex* Rothschild, 1906 [44]
		*Ischnopsyllus (Ischnopsyllus) variabilis* (Wagner, 1898) [44]
		*Nycteridopsylla longiceps* Rothschild, 1908 [29, 37, 167]
		**Trematoda**
		*Allassogonoporus amphoraeformis* (Mödlinger, 1930) [89, 167]
		*Parabascus duboisi* (Hurkova, 1961) [89, 167]
		*Plagiorchis vespertilionis* (O.F. Müller, 1784) [89, 167]
		*Prosthodendrium (Prosthodendrium) hurkovaae* Dubois, 1960 [89]
*Myotis emarginatus* (Geoffroy, 1806)	19 recognised species	**Acari**
	2 innominate species	Absence of *Ixodes canisuga* Johnston, 1849 [28]
		Absence of *Pholeoixodes hexagonus* (Leach, 1815) [28]
		*Argas (Secretargas) transgariepinus* White, 1846 [45, 189]
		*Argas vespertilionis* (Latreille, 1796) [28]
		*Dermanyssus* sp. [86]
		*Eyndhovenia euryalis* (G. Canestrini, 1885) [63, 121]
		*Ixodes (Eschatocephalus) vespertilionis* C.L. Koch, 1844 [28, 32, 38, 136, 137, 177]
		*Spinturnix emarginatus* (Kolenati, 1856) [7, 76, 78]
		**Cestoda**
		*Milina grisea* van Beneden, 1873 [68, 86, 167]
		**Diptera**
		*Nycteribia (Achrocholidia) vexata* Westwood, 1835 ! [23]
		*Nycteribia (Nycteribia) pedicularia* Latreille, 1805 ? [23]
		*Basilia (Basilia) italica* Theodor, 1954 [4, 34]
		*Basilia (Basilia) nana* Theodor & Moscona, 1954 [43, 237]
		*Nycteribia (Nycteribia) latreillii* (Leach, 1817) [22]
		**Hemiptera**
		*Cimex lectularius* Linnaeus, 1758 ? [253, 254]
		*Cimex dissimilis* (Horváth, 1910) [254]
		**Nematoda**
		*Litomosa dogieli* Bogdanov & Vladimirov, 1956 [19]
		*Litomosa filaria* (Beneden, 1873) [86, 167]
		*Litomosa ottavianii* Lagrange & Bettini, 1948 [68, 167]
		*Rictularia* sp. [86]
		**Siphonaptera**
		*Ischnopsyllus (Ischnopsyllus) simplex* Rothschild, 1906 [29, 37, 142]
		**Trematoda**
		*Lecithodendrium linstowi* Dollfus, 1931 [68, 167]
		*Plagiorchis vespertilionis* (O.F. Müller, 1784) [68, 86, 167]
*Myotis myotis* (Borkhausen, 1797)	38 species	**Acari**
	(33 recognised species and 5 invalid species)	Absence of *Ixodes canisuga* Johnston, 1849 [28]
	3 innominate species	Absence of *Pholeoixodes hexagonus* (Leach, 1815) [28]
		[*Dermanyssus murinus* (Lucas, 1840)] [101, 104, 167, 183, 195]
		[*Liponyssus decussatus* (Kolenati) (=*Caris decussata* Kolenati, 1856 ?)] [23]
		[Pteroptus vespertilionis (Dufour, 1832)] [90, 92]
		[*Spinturnix vespertilionis* (C.L. Koch)] [70]
		[*Spinturnix vespertilionis*] [25]
		*Ixodes (Eschatocephalus) simplex simplex* Neumann, 1906! [85]
		*Ichoronyssus* sp. ? [25]
		*Macronyssus rhinolophi* (Oudemans, 1902) ? [152, 212]
		*Argas vespertilionis* (Latreille, 1796) [28]
		*Ixodes (Eschatocephalus) vespertilionis* C.L. Koch, 1844 [23, 28, 32, 38, 154, 166]
		Nycteridocoptes poppei Oudemans, 1898 [24, 113]
		Spinturnix myoti (Kolenati, 1856) [14, 76, 78, 177, 226]
		*Spinturnix psi* (Kolenati, 1856) [76]
		*Ichoronyssus spinosus (*Oudemans, 1902)*/Ichoronyssus scutatus* (Kolenati, 1856) [24, 70, 72]
		*Ixodes* sp. [178]
		*Spinturnix* sp. [133]
		*Steatonyssus* sp. [152, 212]
		**Cestoda**
		*Milina grisea* van Beneden, 1873 [68, 159, 167]
		**Diptera**
		*Nycteribia (Nycteribia) latreillii* (Leach, 1817) [1, 28, 30]
		*Nycteribia (Nycteribia) pedicularia* Latreille, 1805 [25, 186]
		*Nycteribia (Nycteribia) schmidlii* Schiner, 1853 [1, 30]
		*Nycteribia (Achrocholidia) vexata* Westwood, 1835 [1, 23, 25, 28, 30, 38, 43, 167]
		*Penicillidia (Neopenicillidia) conspicua* Speiser, 1901 [25]
		*Penicillidia (Penicillidia) dufourii* (Westwood, 1834) [1, 14, 22, 28, 30, 43, 91, 186, 237, 242]
		**Hemiptera**
		*Cimex dissimilis* (Horváth, 1910) ? [177]
		*Cimex lectularius* Linnaeus, 1758 [253, 254]
		**Nematoda**
		*Rictularia plagiostoma* (Wedl, 1861)! [233]
		*Litomosa dogieli* Bogdanov & Vladimirov, 1956 [19]
		*Litomosa filaria* (Beneden, 1873) [75, 86, 167]
		*Molinostrongylus ornatus* (Monnig, 1927) [68]
		*Molinostrongylus tipula* (Beneden, 1873) [57]
		*Pterygodermatites (Neopaucipectines) bovieri* (Blanchard, 1886) [56, 167, 177, 211, 233, 235, 245]
		**Siphonaptera**
		[*Pulex vespertilionis* Dugès, 1832] [95]
		*Nycteridopsylla longiceps* Rothschild, 1908 ! [228]
		*Ischnopsyllus (Ischnopsyllus) intermedius* (Rothschild, 1898) [2, 29, 33, 37, 142, 167]
		*Ischnopsyllus (Ischnopsyllus) octactenus* (Kolenati, 1856) [25, 29, 37, 167]
		*Ischnopsyllus (Hexactenopsylla) hexactenus* (Kolenati, 1856) [14, 29, 33, 37, 167, 228]
		*Ischnopsyllus (Ischnopsyllus) simplex* Rothschild, 1906 [29, 37, 85]
		*Nycteridopsylla pentactena* (Kolenati, 1856) [29, 37, 167]
		**Trematoda**
		*Lecithodendrium linstowi* Dollfus, 1931 [68, 167]
		*Plagiorchis vespertilionis* (O.F. Müller, 1784) [86]
		*Prosthodendrium (Prosthodendrium) chilostomum* (Mehlis, 1831) [57, 61, 68, 167]
		See also [217, 220]
*Myotis mystacinus* (Leisler *in* Kuhl, 1817)	14 species	**Acari**
	(11 recognised species and 3 invalid species)	Absence of *Ixodes canisuga* Johnston, 1849 [28]
		Absence of *Pholeoixodes hexagonus* (Leach, 1815) [28]
		[*Gamassus dermanyssoïdes* (Mégnin)] [248]
		[*Pteroptus trouessarti* Mégnin (=*Haemomyson trouessarti* (Mégnin)?)] [248]
		[*Pteroptus vespertilionis* (Dufour, 1832)] [248]
		*Ixodes (Eschatocephalus) simplex simplex* Neumann, 1906! [85]
		*Argas (Carios) vespertilionis* (Latreille, 1802) [45]
		*Argas vespertilionis* (Latreille, 1796) [28]
		*Ixodes (Eschatocephalus) vespertilionis* C.L. Koch, 1844 [28, 32, 38, 85]
		*Spinturnix mystacinus* (Kolenati, 1857) [76, 78]
		**Diptera**
		*Basilia (Basilia) italica* Theodor, 1954 [34, 35, 43, 237]
		*Basilia (Basilia) nana* Theodor & Moscona, 1954 [241]
		*Nycteribia (Nycteribia) kolenatii* Theodor & Moscona, 1954 [147, 241]
		**Siphonaptera**
		*Ischnopsyllus (Ischnopsyllus) simplex* Rothschild, 1906 [2, 29, 33, 37, 44, 85?, 142, 156]
		*Nycteridopsylla longiceps* Rothschild, 1908 [29, 37, 38, 167]
		*Nycteridopsylla pentactena* (Kolenati, 1856) [29, 37, 167]
*Myotis nattereri* (Kuhl, 1817)	8 recognised species	**Acari**
		Absence of *Ixodes canisuga* Johnston, 1849 [28]
		Absence of *Pholeoixodes hexagonus* (Leach, 1815) [28]
		*Ixodes (Eschatocephalus) vespertilionis* C.L. Koch, 1844 [28, 32]
		*Spinturnix myoti* (Kolenati, 1856) [63, 121]
		**Diptera**
		*Basilia (Basilia) nattereri* (Kolenati, 1857) [4, 28, 30, 43, 187]
		*Basilia (Basilia) nana* Theodor & Moscona, 1954 [4, 30, 237]
		*Nycteribia (Nycteribia) kolenatii* Theodor & Moscona, 1954 [28, 147]
		**Siphonaptera**
		*Ischnopsyllus (Ischnopsyllus) simplex* Rothschild, 1906 [29, 33, 37, 44, 85, 142]
		*Nycteridopsylla longiceps* Rothschild, 1908 [29, 37, 167]
		*Nycteridopsylla pentactena* (Kolenati, 1856) [29, 37, 167]
*Myotis punicus* Felten, Spitzenberger & Storch, 1977	1 recognised species	Spinturnix myoti (Kolenati, 1856) [64]
*Myotis* sp.	4 recognised species	**Acari**
		*Argas vespertilionis* (Latreille, 1796) [171, 242]
		Spinturnix myoti (Kolenati, 1856) ? [14]
		**Hemiptera**
		*Cimex dissimilis* (Horváth, 1910) [253]
		**Siphonaptera**
		*Ischnopsyllus (Hexactenopsylla) hexactenus* (Kolenati, 1856) [44]
*Nyctalus lasiopterus* (Schreber, 1780)	1 recognised species	**Acari**
		*Pteracarus pipistrellius maximis* Uchikawa, 1989 [251]
*Nyctalus leisleri* (Kuhl, 1817)	2 recognised species	**Acari**
		*Argas (Carios) vespertilionis* (Latreille, 1802) [45]
		**Siphonaptera**
		*Ischnopsyllus (Ischnopsyllus) intermedius* (Rothschild, 1898) [44]
*Nyctalus noctula* (Schreber, 1774)	14 species (8 recognised species and 6 invalid species)	**Acari**
		[*Acarus vespertilionis* Hermann, 1804 - la Mite de la chauve-souris] [135]
		[*Caris vespertilionis* = *Argas vespertilionis* (Latreille, 1796) ?] ? [168, 171, 201]
		[*Dermanyssus coriaceus* Gervais (=*Hirstionyssus arcuatus* (Koch, 1839)?)] [11, 13, 103, 212, 221, 222, 258, 259]
		[*Haemomyson trouessarti* (Mégnin) (=*Leiognathus arcuatus*=*Hirstionyssus arcuatus* (Koch, 1839)? See [244]]
		[*Pteroptus vespertilionis* (Dufour, 1832)] [131, 177]
		[*Pteroptus vespertilionis*] [131]
		*Hirstionyssus arcuatus* (Koch, 1839) [11, 12, 103, 124, 127, 167, 177, 212, 221, 258, 259]
		*Spinturnix helvetiae* (Deunff, Aellen & Keller, 1986) [recorded at Col de Bretolet, at the border between France and Switzerland] [79]
		*Spinturnix acuminatus* (C.L. Koch, 1836) [recorded at Col de Bretolet, at the border between France and Switzerland] [79]
		**Diptera**
		*Nycteribia (Nycteribia) pedicularia* Latreille, 1805![85]
		**Siphonaptera**
		*Ischnopsyllus (Ischnopsyllus) variabilis* (Wagner, 1898) ? [37]
		*Ischnopsyllus (Ischnopsyllus) elongatus* (Curtis, 1832) [37, 42, 44, 167, 204, 228!, 231!]
		*Ischnopsyllus (Ischnopsyllus) intermedius* (Rothschild, 1898) [3, 44]
		*Nycteridopsylla eusarca* Dampf, 1908 [37, 42]
*Pipistrellus kuhlii* (Kuhl, 1817)	9 recognised species	**Acari**
		Absence of *Ixodes canisuga* Johnston, 1849 [28]
		Absence of *Ixodes (Eschatocephalus) simplex simplex* Neumann, 1906 [32]
		Absence of *Pholeoixodes hexagonus* (Leach, 1815) [28]
		*Argas (Carios) vespertilionis* (Latreille, 1802) [45]
		*Argas vespertilionis* (Latreille, 1796) [28, 166]
		*Ixodes (Eschatocephalus) vespertilionis* C.L. Koch, 1844 [28]
		**Siphonaptera**
		*Ischnopsyllus (Hexactenopsylla) hexactenus* (Kolenati, 1856) [29]
		*Ischnopsyllus (Ischnopsyllus) octactenus* (Kolenati, 1856) [29, 33, 37, 167]
		*Ischnopsyllus (Ischnopsyllus) variabilis* (Wagner, 1898) [37, 44, 142]
		*Nycteridopsylla longiceps* Rothschild, 1908 [37, 44, 156]
		**Trematoda**
		*Lecithodendrium linstowi* Dollfus, 1931 [86]
		*Pycnoporus heteroporus* (Dujardin, 1845) [86]
*Pipistrellus nathusii* (Keyserling & Blasius, 1839)	8 recognised species	**Acari**
		Absence of *Ixodes canisuga* Johnston, 1849 [28]
		Absence of *Ixodes (Eschatocephalus) simplex simplex* Neumann, 1906 [32]
		Absence of *Pholeoixodes hexagonus* (Leach, 1815) [28]
		*Acanthophthirius* (Acanthophthirius) poppei (Trouessart, 1895) [52, 102, 114, 167, 249]
		*Argas (Carios) vespertilionis* (Latreille, 1802) [45]
		*Argas vespertilionis* (Latreille, 1796) [28]
		*Ixodes (Eschatocephalus) vespertilionis* C.L. Koch, 1844 [28]
		**Siphonaptera**
		*Ischnopsyllus (Ischnopsyllus) octactenus* (Kolenati, 1856) [29, 33, 37, 167]
		*Ischnopsyllus (Ischnopsyllus) variabilis* (Wagner, 1898) [2, 29, 33, 37, 44]
		*Nycteridopsylla longiceps* Rothschild, 1908 [37, 44, 167]
		*Nycteridopsylla pentactena* (Kolenati, 1856) [44]
*Pipistrellus pipistrellus* (Schreber, 1774)	23 species	**Acari**
	(17 recognised species and 6 invalid species and one species complex)	Absence of *Ixodes canisuga* Johnston, 1849 [28]
		Absence of *Ixodes (Eschatocephalus) simplex simplex* Neumann, 1906 [32]
		Absence of *Pholeoixodes hexagonus* (Leach, 1815) [28]
		Absence of [*Pteroptus vespertilionis*] [67]
		[*Argas caris*] [191]
		[*Caris vespertilionis* = *Argas vespertilionis* (Latreille, 1796) ?] ? [258]
		[*Gamasus pteroptoides* (Mégnin)] [190]
		[*Haemomyson trouessarti* (Mégnin) (=*Leiognathus arcuatus*=*Hirstionyssus arcuatus* (Koch, 1839)? See [221]]
		[*Laelaps (Iphis) agilis* Koch, Berlese = *Gamasus pteroptoides* (Mégnin)? = *Laelaps agilis* C.L. Koch, 1836 ?] ([244], see also [190])
		[*Pteroptus vespertilionis* Hermann] [215]
		*Argas (Carios) vespertilionis* (Latreille, 1802) [31, 45, 177, 239]
		*Argas vespertilionis* (Latreille, 1796) [18, 28, 38, 67, 166]
		*Ixodes (Eschatocephalus) vespertilionis* C.L. Koch, 1844 [28, 86, 199]
		See also [201]
		**Diptera**
		*Nycteribia (Nycteribia) pedicularia* Latreille, 1805 ? [214, 215]
		*Basilia (Basilia) mediterranea* Hůrka, 1970 [40, 43, 167]
		*Nycteribia (Nycteribia) kolenatii* Theodor & Moscona, 1954 [241]
		*Nycteribia (Nycteribia) schmidlii* Schiner, 1853 [147]
		*Nycteribia* sp. - Complex of species *Nycteribia kolenatii/latreillii/pedicularia* [177, 214, 215]
		**Siphonaptera**
		*Ischnopsyllus (Ischnopsyllus) octactenus* (Kolenati, 1856) [2, 29, 33, 36, 37, 44, 142, 164, 167, 177, 227]
		*Ischnopsyllus (Ischnopsyllus) variabilis* (Wagner, 1898) [33, 37, 85]
		*Nycteridopsylla ancyluris ancyluris* Jordan, 1942 [33, 37, 44, 142, 157, 164]
		*Nycteridopsylla longiceps* Rothschild, 1908 [2, 29, 33, 37, 85, 167]
		*Nycteridopsylla pentactena* (Kolenati, 1856) [29, 37, 38, 167]
		**Trematoda**
		*Lecithodendrium linstowi* Dollfus, 1931 [68, 167]
		*Parabascus semisquamosus* (Braun, 1900) [60, 68, 167]
		*Plagiorchis vespertilionis* (O.F. Müller, 1784) [68, 86, 167]
		*Pycnoporus heteroporus* (Dujardin, 1845) [57, 61, 68, 97, 163, 167, 235]
*Pipistrellus* sp.	3 recognised species	**Acari**
Nota bene: according to Lord *et al.,* for studies published before the 1990’s, *Pipistrellus pipistrellus* and *Pipistrellus* sp. specimens should be treated as potentially also including *P. pygmaeus.*		*Argas (Carios) vespertilionis* (Latreille, 1802) [45]
		**Diptera**
		*Basilia (Basilia) mediterranea* Hůrka, 1970 [41]
		*Penicillidia (Penicillidia) dufourii* (Westwood, 1834) [53]
*Plecotus auritus* (Linné, 1758)	16 recognised species	**Acari**
	(15 recognised species and 1 invalid species)	Absence of *Ixodes canisuga* Johnston, 1849 [28]
	1 innominate species	Absence of *Ixodes (Eschatocephalus) simplex simplex* Neumann, 1906 [32]
		Absence of *Pholeoixodes hexagonus* (Leach, 1815) [28]
		[*Spinturnix vespertilionis*] [70]
		*Argas (Carios) vespertilionis* (Latreille, 1802) [45]
		*Argas vespertilionis* (Latreille, 1796) [28]
		*Ixodes (Eschatocephalus) vespertilionis* C.L. Koch, 1844 [28, 199]
		*Spinturnix plecotina* (C.L. Koch, 1839) [63, 76, 167]
		See also [201]
		**Cestoda**
		*Vampirolepis* sp*./Milina* sp. [86]
		**Diptera**
		*Basilia (Basilia) nana* Theodor & Moscona, 1954 [28, 237]
		*Basilia (Basilia) nattereri* (Kolenati, 1857) [28, 187]
		*Phthiridium biarticulatum* Hermann, 1804 [85, 86, 237]
		**Nematoda**
		*Litomosa filaria* (Beneden, 1873) [19, 167]
		*Seuratum mucronatum* (Rudolphi, 1809) [54, 86, 194]
		**Siphonaptera**
		*Ischnopsyllus (Ischnopsyllus) simplex* Rothschild, 1906 ? [29, 37]
		*Ischnopsyllus (Ischnopsyllus) variabilis* ! (Wagner, 1898) [85]
		*Ischnopsyllus (Hexactenopsylla) hexactenus* (Kolenati, 1856) [2, 33, 37, 44, 142, 177, 227, 228]
		*Nycteridopsylla longiceps* Rothschild, 1908 [33, 37, 85, 167, 227]
		*Nycteridopsylla pentactena* (Kolenati, 1856) [29, 33, 37, 167]
		**Trematoda**
		*Plagiorchis vespertilionis* (O.F. Müller, 1784) [86, 167]
		*Prosthodendrium* sp. [86]
*Plecotus austriacus* Fischer, 1829	7 recognised species	**Acari**
		Absence of *Ixodes (Eschatocephalus) simplex simplex* Neumann, 1906 [32]
		*Argas (Carios) vespertilionis* (Latreille, 1802) [31]
		*Argas (Secretargas) transgariepinus* White, 1846 [45, 189]
		*Spinturnix plecotina* (C.L. Koch, 1839) [63, 76, 167]
		See also [128]
		**Siphonaptera**
		*Ischnopsyllus (Hexactenopsylla) hexactenus* (Kolenati, 1856) [33, 37, 44]
		*Nycteridopsylla pentactena* (Kolenati, 1856) [33, 37, 167]
		**Trematoda**
		*Lecithodendrium linstowi* Dollfus, 1931 [68, 167]
		*Parabascus lepidotus* Looss, 1907 [68, 167]
*Plecotus* sp.	1 recognised species	**Nematoda**
		*Seuratum mucronatum* (Rudolphi, 1809) [97]
*Rhinolophus euryale* Blasius, 1853	21 recognised species	**Acari**
		Absence of *Ixodes canisuga* Johnston, 1849 [28]
		Absence of *Pholeoixodes hexagonus* (Leach, 1815) [28]
		*Eyndhovenia euryalis* (G. Canestrini, 1885) [50, 63, 70, 76, 78, 105, 139, 167, 235]
		*Ichoronyssus scutatus* (Kolenati, 1856) [50, 65, 167, 205, 212, 235, 246]
		*Ixodes (Eschatocephalus) vespertilionis* C.L. Koch, 1844 [28, 32, 85, 166]
		*Macronyssus granulosus* (Kolenati, 1856) [51, 167]
		*Neomyobia slovenica* Dusbábek, 1969 [50–52, 167]
		*Neotrombicula vandeli* Kolebinova & Vercammen-Grandjean, 1971 [7, 162, 167]
		*Paraperiglischrus rhinolophinus* (C.L. Koch, 1841) [50, 76, 138, 167, 235]
		*Riedlinia (Riedlinia) petarberoni* (Kolebinova & Vercammen-Grandjean, 1970) [7, 51, 161, 167]
		*Sasatrombicula* (*Sasatrombicula*) *hexasternalae* (Vercammen-Grandjean, 1963) [51, 162, 167]
		*Spinturnix myoti* (Kolenati, 1856) [76]
		*Spinturnix psi* (Kolenati, 1856) [76]
		**Diptera**
		*Nycteribia (Nycteribia) pedicularia* Latreille, 1805 ! [85]
		*Nycteribia (Achrocholidia) vexata* Westwood, 1835! [85]
		*Phthiridium biarticulatum* Hermann, 1804 [28, 30, 38, 115, 167, 242]
		**Hemiptera**
		*Cimex lectularius* Linnaeus, 1758? [253, 254]
		**Nematoda**
		*Litomosa ottavianii* Lagrange & Bettini, 1948 [68, 167]
		*Riouxgolvania rhinolophi* (Bain & Chabaud, 1968) [20, 167]
		**Siphonaptera**
		*Rhinolophopsylla unipectinata unipectinata* (Taschenberg, 1880) [2, 29, 33, 142, 167]
		**Trematoda**
		*Lecithodendrium linstowi* Dollfus, 1931 [68, 167]
		*Parabascus lepidotus* Looss, 1907 [68, 167]
		*Plagiorchis vespertilionis* (O.F. Müller, 1784) [68, 167]
*Rhinolophus ferrumequinum* (Schreber, 1774)	50 species	**Acari**
	(44 recognised species and 6 invalid species)	Absence of *Ixodes canisuga* Johnston, 1849 [28]
	2 innominate species	Absence of *Pholeoixodes hexagonus* (Leach, 1815) [28]
		[*Haemogamasus setosus* (Kolenati)] [5]
		[*Liponyssus arcuatus* Koch (=*Hirstionyssus arcuatus* (C.L. Koch, 1839) (*pro parte*) et *Steatonyssus murinus* (Lucas, 1840) (*pro parte*)] [200]
		[*Pteroptus vespertilionis* Hermann] ([215], see also [177])
		[*Pteroptus vespertilionis* (Dufour, 1832)] [200]
		*Macronyssus rhinolophi* (Oudemans, 1902) ? [152, 212]
		*Ixodes (Eschatocephalus) simplex simplex* Neumann, 1906! [85]
		*Macronyssus ellipticus* (Kolenati, 1856) ! [152, 177, 212]
		According to Radovsky [212], this observation published by Husson & Daum [152] is dubious.
		*Macronyssus longimanus* (Kolenati) ! [152, 177, 212]: according to Radovsky [212], this observation published by Husson & Daum [152] is dubious.
		*Alabidocarpus diceratops* Lawrence, 1952 [5, 110, 167]
		*Alabidocarpus megalonyx* (Trouessart, 1895) [52, 111, 167, 208, 218, 249]
		*Alabidocarpus minor* (Rollinat & Trouessart, 1897) [52, 111, 167, 208, 218, 249]
		*Argas (Carios) vespertilionis* (Latreille, 1802) [45]
		*Eyndhovenia euryalis* (G. Canestrini, 1885) [50, 63, 76, 78, 167]
		*Eyndhovenia euryalis oudemansi* (Eyndhoven, 1941) [18, 105, 252]
		*Ixodes (Eschatocephalus) vespertilionis* C.L. Koch, 1844 [28, 32, 38, 45, 51, 58, 154, 167, 177, 199, 200, 214, 215, 218, 229, 239]
		*Labidocarpus (Labidocarpus) rollinati* Trouessart, 1895 [167, 218, 232]
		*Neomyobia rollinati* (Poppe, 1908) [50, 52, 167]
		*Notoedres (Notoedres) chiropteralis* (Trouessart, 1896) [106, 109, 167, 218, 250]
		*Nycteridocoptes eyndhoveni* Fain, 1959 [49, 107]
		*Paraperiglischrus rhinolophinus* (C.L. Koch, 1841) [50, 78, 167, 235]
		*Psorergates rhinolophi* Fain, 1959 [108, 167]
		*Sasatrombicula (Sasatrombicula) hexasternalae* (Vercammen-Grandjean, 1963) [51, 162, 167]
		*Spinturnix psi* (Kolenati, 1856) [139, 223]
		*Steatonyssus spinosus* Willmann, 1936 [50, 51, 167]
		See also [201]
		**Cestoda**
		*Hymenolepis* sp. [86]
		*Milina grisea* van Beneden, 1873 [68, 71, 159, 196, 200, 243]
		**Diptera**
		[*Nycteribia vespertilionis* Latreille] [73]
		*Nycteribia (Achrocholidia) vexata* Westwood, 1835 ! [85]
		*Penicillidia (Penicillidia) dufourii* (Westwood, 1834) ? [200]
		*Brachytarsina flavipennis* Macquart, 1851 [26, 40, 43, 117, 155, 237]
		*Nycteribia (Nycteribia) schmidlii* Schiner, 1853 [1]
		*Nycteribia* sp. [73, 74]
		*Phthiridium biarticulatum* Hermann, 1804 [1, 28, 30, 43, 85, 115, 126, 135, 151, 152, 154, 156, 175, 177, 237, 242]
		**Hemiptera**
		*Cimex dissimilis* (Horváth, 1910) [254]
		*Cimex lectularius* Linnaeus, 1758 [253, 254]
		**Nematoda**
		*Litomosa ottavianii* Lagrange & Bettini, 1948 [19, 167]
		*Strongylacantha glycirrhyza* van Beneden, 1873 [68, 167, 200, 243]
		*Trichosomum speciosum* van Beneden, 1873 [200]
		See also [174]
		**Siphonaptera**
		*Ischnopsyllus (Ischnopsyllus) simplex* Rothschild, 1906 ?[152, 177]
		*Ischnopsyllus (Ischnopsyllus) intermedius* (Rothschild, 1898) [29]
		*Ischnopsyllus (Hexactenopsylla) hexactenus* (Kolenati, 1856) [152]
		*Nycteridopsylla pentactena* (Kolenati, 1856) [29, 37, 152, 164, 167, 177]
		*Rhinolophopsylla unipectinata unipectinata* (Taschenberg, 1880) [2, 33, 38, 44, 142, 152, 156, 164, 167, 177, 227, 242]
		**Trematoda**
		[*Paralecithodendrium chilostomum* (Mehlis) (=*Prosthodendrium (Prosthodendrium) chilostomum* (Mehlis, 1831)?)] [200]
		*Lecithodendrium granulosum* Looss, 1907 ? [200]
		*Lecithodendrium linstowi* Dollfus, 1931 [68, 167, 243]
		*Lecithodendrium moedlingeri* (Pande, 1935) [243]
		*Mesotretes peregrinus* (Braun, 1900) [68, 71, 88, 167, 188, 243]
		*Parabascus lepidotus* Looss, 1907 [68, 167]
		*Plagiorchis vespertilionis* (O.F. Müller, 1784) [68, 71, 86–88, 167, 200, 243]
		*Prosthodendrium (Prosthodendrium) carolinum* Hurková, 1959 [167, 243]
		*Prosthodendrium (Prosthodendrium) chilostomum* (Mehlis, 1831) [200, 243]
		*Prosthodendrium (Prosthodendrium) longiforme* (Bhalerao, 1926) [71, 167, 243]
		See also [174]
*Rhinolophus hipposideros* (Bechstein, 1800)	21 species (19 recognised species and 2 invalid species)	**Acari**
	2 innominate species	Absence of *Ixodes canisuga* Johnston, 1849 [28]
		Absence of *Pholeoixodes hexagonus* (Leach, 1815) [28]
		[*Pteroptus vespertilionis* (Dufour, 1832)] [200]
		*Ixodes* sp. [178]
		*Ixodes (Eschatocephalus) vespertilionis* C.L. Koch, 1844 [23, 28, 32, 152, 154, 199, 200]
		*Labidocarpus (Labidocarpus) rollinati* Trouessart, 1895 ([167, 218] see also [52, 208])
		*Leptotrombidium russicum* (Oudemans, 1902) [161]
		*Macronyssus rhinolophi* (Oudemans, 1902) [50, 152, 167, 212]
		*Neomyobia chiropteralis chiropteralis* (Michael, 1884) [50, 52, 100, 114, 167]
		*Paraperiglischrus rhinolophinus* (C.L. Koch, 1841) [50, 138, 167, 223, 235]
		*Sasatrombicula (Sasatrombicula) bureschi* Kolebinova & Beron, 1965 [51, 52, 162, 167]
		*Sasatrombicula (Sasatrombicula) hexasternalae* (Vercammen-Grandjean, 1963) [51, 162, 167]
		**Anoplura**
		*Polyplax serrata* (Burmeister, 1839) ! [17, 177, 216]
		**Cestoda**
		*Milina grisea* van Beneden, 1873 ? [200]
		**Diptera**
		*Nycteribia (Achrocholidia) vexata* Westwood, 1835! [85]
		*Nycteribia (Nycteribia) pedicularia* Latreille, 1805 ? [200]
		*Phthiridium biarticulatum* Hermann, 1804 [115, 126?, 135?, 167, 237]
		**Nematoda**
		*Trichosomum speciosum* van Beneden, 1873 [200]
		See also [174]
		**Siphonaptera**
		*Rhinolophopsylla unipectinata unipectinata* (Taschenberg, 1880) [2, 86, 142, 167, 200, 228]
		**Trematoda**
		*Lecithodendrium granulosum* Looss, 1907? [200]
		[*Paralecithodendrium chilostomum* (Mehlis) (=*Prosthodendrium (Prosthodendrium) chilostomum* (Mehlis, 1831)?)] [200]
		See also [174]
		*Lecithodendrium* sp. [86]
		*Mesotretes peregrinus* (Braun, 1900) [68, 167, 188]
		*Plagiorchis vespertilionis* (O.F. Müller, 1784) [68, 86, 167, 200]
		*Prosthodendrium (Prosthodendrium) chilostomum* (Mehlis, 1831) [86, 167, 200, 235]
*Rhinolophus mehelyi* Matschie, 1901	1 recognised species	**Diptera**
		*Nycteribia (Nycteribia) schmidlii* Schiner, 1853 [147]
*Rhinolophus* sp.	5 species(4 recognised species and 1 invalid species)	**Acari**
	1 innominate species	*Eyndhovenia euryalis* (G. Canestrini, 1885) [51, 76]
		*Ixodes (Eschatocephalus) vespertilionis* C.L. Koch, 1844 [5, 58, 126, 197]
		[*Liponyssus setosus* (Kolenati)] [72]
		*Neomyobia* sp. [126]
		**Nematoda**
		*Litomosa ottavianii* Lagrange & Bettini, 1948 [19, 167]
		**Siphonaptera**
		*Rhinolophopsylla unipectinata unipectinata* (Taschenberg, 1880) [44]
*Tadarida teniotis* (Rafinesque, 1814)	1 recognised species	**Siphonaptera**
		*Araeopsylla gestroi* (Rothschild, 1906) [3, 37, 44, 85]

A large host group comprising the following taxa was identified with Acari infections: *Barbastella barbastellus, Eptesicus serotinus*, *Eptesicus* sp., *Hypsugo savii*, *Miniopterus schreibersii*, *Myotis bechsteinii*, *M. blythii*, *M. blythii oxygnathus*, *M. capaccinii, M. dasycneme*, *M. daubentonii*, *M. emarginatus*, *M. myotis*, *M. mystacinus*, *M. nattereri*, *M. punicus, Myotis* sp., *Nyctalus lasiopterus, N. leisleri*, *N. noctula*, *Pipistrellus kuhlii, P. nathusii*, *P. pipistrellus*, *Pipistrellus* sp., *Plecotus auritus*, *P. austriacus*, *Rhinolophus euryale, R. ferrumequinum*, *R. hipposideros* and *Rhinolophus* sp. (see Tables 1 and 2). The oldest works dealing with Acari parasitising bats in France are Geoffroy’s *Histoire abrégée des insectes* [123] and Latreille’s *Précis des caractères génériques des insectes, disposés dans un ordre naturel* [168], dated 1762 and 1797. Geoffroy mentioned the tick (found on an unidentified bat) as *Acarus fuscus ovatus, pedibulus pallidis, vespertilionis,* a taxon treated as *Caris vespertilionis* by Lamarck in the *Histoire naturelle des animaux sans vertèbres* (1839). The book of Latreille contains an observation of *Carios* on “la Chauve-Souris noctule” (=*Nyctalus noctula* or *Nyctalus* sp.). This taxon is very likely *Argas (Carios) vespertilionis* (Latreille, 1802). Descriptions of some species and subspecies are based on type material from France (e.g. *Spinturnix nobleti* [81], *S. bechsteini* [82], *Pteracarus pipistrellius maximis* [251], *Myobia poppei* (= *Acanthophthirius (Acanthophthirius) poppei*) [249] and *Spinturnix andegavinus* [76]). The majority of published data on parasites of chiropteran populations in France deal with Arachnida and similar findings were noted by Lanza [167], Krištofík & Danko [165] and Frank *et al.* [121] in Italy, Slovakia and other European countries.

**Table 2:** List of bat parasites (*Acari, Anoplura, Cestoda, Diptera, Hemiptera, Nematoda, Siphonaptera, Trematoda*) and their hosts in France (including Corsica), based on the published literature, with reported synonyms. Authors are listed in the bibliography. See also the work entitled *Les parasites métazoaires des Chiroptères de France (Acari, Anoplura, Cestoda, Diptera, Hemiptera, Nematoda, Siphonaptera, Trematoda) : contribution à un état des lieux bibliographique (1762-2018) et à l’établissement d’une liste nationale* (2019). Invalid species are listed in brackets. Records marked with an exclamation mark (!) are invalid. Records marked with a question mark (?) are dubious. They may require further clarification.

Parasite species	Number of reported hosts	Bat species and citation
**Acari**		
**Acari, part 1/3: generally recognised taxa (*n* = 53), with their reported synonyms, and records without identification to species level (*n* = 6)**		
*Acanthophthirius (Myotimyobia) pantopus (Poppe et Trouessart, 1895)*	1	*Barbastella barbastellus* (Schreber, 1774) [102, 114, 167, 235, 249]
*Acanthophthirius (Acanthophthirius) poppei (Trouessart, 1895)*	1	*Pipistrellus nathusii* (Keyserling & Blasius, 1839) [52, 102, 114, 167, 249]
*Alabidocarpus diceratops* Lawrence, 1952	1	*Rhinolophus ferrumequinum* (Schreber, 1774) [5, 110, 167]
*Alabidocarpus megalonyx* (Trouessart, 1895)	1	*Rhinolophus ferrumequinum* (Schreber, 1774) [52, 111, 167, 208, 218, 249]
*Alabidocarpus minor* (Rollinat & Trouessart, 1897)	1	*Rhinolophus ferrumequinum* (Schreber, 1774) [52, 111, 167, 208, 218, 249]
*Argas pipistrellae* (Audouin, 1832) = *Argas vespertilionis* (Latreille, 1796)		
			*Argas transgariepinus* White, 1846 = *Argas (Secretargas) transgariepinus* White, 1846		
*Argas vespertilionis* (Latreille, 1796)	11	*Barbastella barbastellus* (Schreber, 1774) [28]			
		*Eptesicus serotinus* (Schreber, 1774) [28, 38]
		*Miniopterus schreibersii* (Natterer *in* Kuhl, 1817)? [85]
		*Myotis emarginatus* (Geoffroy, 1806) [28]
		*Myotis myotis* (Borkhausen, 1797) [28]
		*Myotis mystacinus* (Leisler *in* Kuhl, 1817) [28]
		*Myotis* sp. [171?, 242]
		*Pipistrellus kuhlii* (Kuhl, 1817) [28, 166]
		*Pipistrellus nathusii* (Keyserling & Blasius, 1839) [28]
		*Pipistrellus pipistrellus* (Schreber, 1774) [18, 28, 38, 67, 166]
		*Plecotus auritus* (Linné, 1758) [28]
*Argas (Carios) vespertilionis* (Latreille, 1802)	12	*Eptesicus serotinus* (Schreber, 1774) [31, 45, 86]
		*Hypsugo savii* (Bonaparte, 1837) [45]
		*Myotis dasycneme* (Boie, 1825) [31]
		*Myotis mystacinus* (Leisler *in* Kuhl, 1817) [45]
		*Nyctalus leisleri* (Kuhl, 1817) [45]
		*Pipistrellus kuhlii* (Kuhl, 1817) [45]
		*Pipistrellus nathusii* (Keyserling & Blasius, 1839) [45]
		*Pipistrellus pipistrellus* (Schreber, 1774) [31, 45, 177, 239]
		*Pipistrellus* sp. [45]
		*Plecotus auritus* (Linné, 1758) [45]
		*Plecotus austriacus* Fischer, 1829 [31]
		*Rhinolophus ferrumequinum* (Schreber, 1774) [45]
		See also [5, 167–169]
*Argas (Secretargas) transgariepinus* White, 1846	5	Chiroptera gen. sp. [189]
		*Eptesicus serotinus* (Schreber, 1774) [45, 189]
		*Hypsugo savii* (Bonaparte, 1837) [45, 189]
		*Myotis emarginatus* (Geoffroy, 1806) [45, 189]
		*Plecotus austriacus* Fischer, 1829 [45, 189]
		See also [31, 167]
*Chiroptella muscae* (Oudemans, 1906) = *Oudemansidium musca* (Oudemans, 1906)		
			*Calcarmyobia dusbabeki* Uchikawa, 1985?	1?	*Miniopterus schreibersii* (Natterer *in* Kuhl, 1817) [50, 51, 167]
There is only one case. According to Lanza (1999) [publication n°^167^], this field data published by Beron (1971) [publication n°^50^] is doubtful. It could be *Calcarmyobia dusbabeki* Uchikawa, 1985 / *Calcarmyobia rhinolophia* (Radford, 1940).				
*Calcarmyobia rhinolophia* (Radford, 1940)!	1	*Miniopterus schreibersii* (Natterer *in* Kuhl, 1817) [50, 51, 167]
		There is only one case. According to Lanza (1999) [publication n°^167^], this field data published by Beron (1971) [publication n°^50^] is doubtful. It could be *Calcarmyobia dusbabeki* Uchikawa, 1985 / *Calcarmyobia rhinolophia* (Radford, 1940).
*Dermanyssus coriaceus* = *Hirstionyssus arcuatus* (Koch, 1839). See [11, 13, 221, 222]		
*Dermanyssus* sp.	1	*Myotis emarginatus* (Geoffroy, 1806) [86]
*Dermanyssus pipistrellae* (Gervais, 1841) = *Hirstionyssus arcuatus* (Koch, 1839)		
*Diplostaspis daubentonii* Kolenati, 1857 = *Spinturnix andegavinus* (Deunff, 1977) according to Deunff (1977) [76]		
*Diplostaspis stellata* Kolenati, 1859 = *Spinturnix andegavinus* (Deunff, 1977) according to Deunff (1977) [76]		
*Eschatocephalus flavipes* (Koch) = *Ixodes (Eschatocephalus) vespertilionis* C.L. Koch, 1844		
			*Eschatocephalus vespertilionis* C.L. Koch = *Ixodes (Eschatocephalus) vespertilionis* C.L. Koch, 1844		
*Eschatocephalus vespertilionis* (Koch 1844) = *Ixodes (Eschatocephalus) vespertilionis* C.L. Koch, 1844					
			*Eyndhovenia euryalis* (G. Canestrini, 1885)	5	*Miniopterus schreibersii* (Natterer *in* Kuhl, 1817) [76]
*Myotis emarginatus* (Geoffroy, 1806) [63, 121]				
*Rhinolophus euryale* Blasius, 1853 [50, 63, 70, 76, 78, 105, 139, 167, 235]				
*Rhinolophus ferrumequinum* (Schreber, 1774) [50, 63, 76, 78, 167]				
*Rhinolophus* sp. [51, 76]				
*Eyndhovenia euryalis oudemansi* (Eyndhoven, 1941)	1	*Rhinolophus ferrumequinum* (Schreber, 1774) [18?, 105, 252]
*Haemalastor vespertilionis = Ixodes (Eschatocephalus) vespertilionis* C.L. Koch, 1844		
*Hirstionyssus arcuatus* (Koch, 1839)	1	*Nyctalus noctula* (Schreber, 1774) [11, 12, 103, 124, 127, 167, 177, 212, 221, 258, 259]
*Ichoronyssus diversipilis* Vitzthum, 1920	1	*Miniopterus schreibersii* (Natterer *in* Kuhl, 1817) [24]
*Ichoronyssus scutatus* (Kolenati, 1856)	1	*Rhinolophus euryale* Blasius, 1853 [50, 65, 167, 205, 212, 235, 246]
*Ichoronyssus spinosus* (Oudemans) 1902)	1	*Miniopterus schreibersii* (Natterer *in* Kuhl, 1817) [24, 70, 72]
*Ichoronyssus spinosus* (Oudemans, 1902) / *Ichoronyssus scutatus* (Kolenati, 1856)	1	*Myotis myotis* (Borkhausen, 1797)? [24, 70, 72]
*Ichoronyssus* sp.	1	*Myotis myotis* (Borkhausen, 1797)? [25]
*Ixodes chiropterum* Babos et Janisch, 1958 = *Ixodes (Eschatocephalus) simplex simplex* Neumann, 1906		
*Ixodes gracilipes = Ixodes (Eschatocephalus) vespertilionis* C.L. Koch, 1844 ?		
*Ixodes hexagonus* Leach, 1815 = *Pholeoixodes hexagonus* (Leach, 1815)		
*Ixodes longipes* (Lucas, 1872) = *Ixodes (Eschatocephalus) vespertilionis* C.L. Koch, 1844		
*Ixodes (Pomerantzevella) simplex* Neumann 1906 = *Ixodes (Eschatocephalus) simplex simplex* Neumann, 1906		
*Ixodes pospelovae* Emtchuck, 1955 = *Ixodes (Eschatocephalus) simplex simplex* Neumann, 1906		
*Ixodes siculifer* Mégnin, 1880 = *Ixodes (Eschatocephalus) vespertilionis* C.L. Koch, 1844		
*Ixodes reduvius* Geer = *Ixodes (Ixodes) ricinus* (Linnaeus, 1758)		
*Ixodes (Eschatocephalus) vespertilionis* C.L. Koch, 1844	18	*Barbastella barbastellus* (Schreber, 1774) [28, 32, 200]
		Chiroptera gen. sp. [45, 69, 160, 177, 197–199, 248]
		*Eptesicus serotinus* (Schreber, 1774) [28, 32, 166]
		*Miniopterus schreibersii* (Natterer *in* Kuhl, 1817) ([6, 28, 176!] see Beaucournu, 1966, p. 498; paper n°30 [200, 242])
		*Myotis bechsteinii* (Leisler *in* Kuhl, 1817) [28, 32, 38]
		*Myotis daubentonii* (Leisler *in* Kuhl, 1817) [28, 32]
		*Myotis emarginatus* (Geoffroy, 1806) [28, 32, 38, 136, 137, 177]
		*Myotis myotis* (Borkhausen, 1797) [23, 28, 32, 38, 154, 166]
		*Myotis mystacinus* (Leisler *in* Kuhl, 1817) [28, 32, 38, 85]
		*Myotis nattereri* (Kuhl, 1817) [28, 32]
		*Pipistrellus kuhlii* (Kuhl, 1817) [28]
		*Pipistrellus nathusii* (Keyserling & Blasius, 1839) [28]
		*Pipistrellus pipistrellus* (Schreber, 1774) [28, 86, 199]
		*Plecotus auritus* (Linné, 1758) [28, 199]
		*Rhinolophus euryale* Blasius, 1853 [28, 32, 85, 166]
		*Rhinolophus ferrumequinum* (Schreber, 1774) [28, 32, 38, 45, 51, 58, 154, 177, 199, 200, 214, 215, 218, 229, 239]
		*Rhinolophus hipposideros* (Bechstein, 1800) [23, 28, 32, 152, 154, 199, 200]
		*Rhinolophus* sp. [5, 58, 126, 197]
		See also [14, 31, 59, 143, 167, 201, 213]
*Ixodes (Eschatocephalus) simplex simplex* Neumann, 1906	4	Absence in *Myotis blythii* (Tomes, 1857) and *M. blythii oxygnathus* Monticelli, 1885 [32]
		Absence in *Pipistrellus kuhlii* (Kuhl, 1817) [32]
		Absence in *Pipistrellus nathusii* (Keyserling & Blasius, 1839) [32]
		Absence in *Pipistrellus pipistrellus* (Schreber, 1774) [32]
		Absence in *Plecotus auritus* (Linné, 1758) [32]
		Absence in *Plecotus austriacus* Fischer, 1829 [32]

		*Rhinolophus ferrumequinum* (Schreber, 1774) ! [85]
		*Myotis mystacinus* (Leisler *in* Kuhl, 1817) ! [85]
		*Myotis myotis* (Borkhausen, 1797) ! [85]

		*Miniopterus schreibersii* (Natterer *in* Kuhl, 1817) ([15, 31, 32, 45, 51, 143?, 166, 167, 176] see Beaucournu [1966; p. 498] paper n°30)

		See also [213]
*Ixodes* sp.	2	*Myotis myotis* (Borkhausen, 1797) [178]
		*Rhinolophus hipposideros* (Bechstein, 1800) [178]
*Labidocarpus megalonyx* (Trouessart, 1895) = *Alabidocarpus megalonyx* (Trouessart, 1895)		
			*Labidocarpus minor* (Rollinat & Trouessart, 1897) = *Alabidocarpus minor* (Rollinat & Trouessart, 1897)		
*Labidocarpus (Labidocarpus) rollinati* Trouessart, 1895	2	*Rhinolophus ferrumequinum* (Schreber, 1774) [167, 218, 232]			
		*Rhinolophus hipposideros* (Bechstein, 1800) [167, 218] see also [52, 191]
*Leiognathus arcuatus = Hirstionyssus arcuatus* (Koch, 1839)		
			*Leiognathus uncinatus = Macronyssus uncinatus* (Canestrini, 1885)		
*Leptotrombidium (Leptotrombidium) russicum* (Oudemans, 1902) = *Leptotrombidium russicum* (Oudemans, 1902)					
			*Leptotrombidium russicum* (Oudemans, 1902)	2	*Myotis daubentonii* (Leisler *in* Kuhl, 1817) [122, 167, 177, 256] see also [10]
*Rhinolophus hipposideros* (Bechstein, 1800) [161]				
*Leptus autumnalis = Neotrombicula autumnalis* (Shaw, 1790)		
			*Liponyssus ellipticus = Macronyssus ellipticus* (Kolenati, 1856)		
*Liponyssus euryale* Canestrini = *Macronyssus rhinolophi* (Oudemans, 1902)					
			*Liponyssus longimanus = Macronyssus longimanus* (Kolenati)		
*Liponyssus spinosus* Oudemans, 1902 = *Ichoronyssus scutatus* (Kolenati, 1856)					
*Macronyssus ellipticus* (Kolenati, 1856) !	1	*Rhinolophus ferrumequinum* (Schreber, 1774) ! [152, 177, 212]
According to Radovsky [212], this observation published by Husson & Daum [152] is dubious.		
*Macronyssus granulosus* (Kolenati, 1856)	2	*Miniopterus schreibersii* (Natterer *in* Kuhl, 1817) [50, 167]
		*Rhinolophus euryale* Blasius, 1853 [51, 167]
*Macronyssus longimanus* (Kolenati) !	1	*Rhinolophus ferrumequinum* (Schreber, 1774) ! [152, 177, 212]
According to Radovsky [212], this observation published by Husson & Daum [152] is dubious.		
*Macronyssus uncinatus* (Canestrini, 1885)	1	Chiroptera gen. sp. [160, 177]
*Macronyssus rhinolophi* (Oudemans, 1902)	3	*Myotis myotis* (Borkhausen, 1797)? [152?, 212?]
		*Rhinolophus ferrumequinum* (Schreber, 1774)? [152?, 212?]
		*Rhinolophus hipposideros* (Bechstein, 1800) [50, 152?, 167, 212?]
*Macronyssus spinosus* (Oudemans, 1902) = *Ichoronyssus scutatus* (Kolenati, 1856)		
			*Myobia pantopus* = *Acanthophthirius (Myotimyobia) pantopus (Poppe et Trouessart, 1895)*		
*Myobia poppei* = *Acanthophthirius (Acanthophthirius) poppei (Trouessart, 1895)*					
			*Neomyobia chiropteralis chiropteralis* (Michael, 1884)	1	*Rhinolophus hipposideros* (Bechstein, 1800) [50, 52, 100, 167], see also [114]

See also [112]				
*Neomyobia rollinati* (Poppe, 1908)	1	*Rhinolophus ferrumequinum* (Schreber, 1774) [50, 52, 167]
*Neomyobia slovenica* Dusbábek, 1969	1	*Rhinolophus euryale* Blasius, 1853 [50–52, 167]
*Neomyobia* sp.	1	*Rhinolophus* sp. [126]
*Neotrombicula autumnalis* (Shaw, 1790)	2	Chiroptera gen. sp. [62]
		*Myotis daubentonii* (Leisler *in* Kuhl, 1817) [8–10, 122, 167, 177, 256]
*Neotrombicula racovitzai* Feider, 1970	1	*Eptesicus* sp. [5, 118, 167]
*Neotrombicula vandeli* Kolebinova & Vercammen-Grandjean, 1971	1	*Rhinolophus euryale* Blasius, 1853 [7, 162, 167]
*Notoedres (Notoedres) chiropteralis* (Trouessart, 1896)	2	*Rhinolophus ferrumequinum* (Schreber, 1774) [106, 109, 167, 218, 250]
		*Eptesicus serotinus* (Schreber, 1774) [48, 106, 167, 250]
*Nycteridocoptes eyndhoveni* Fain, 1959	1	*Rhinolophus ferrumequinum* (Schreber, 1774) [49, 107]
*Nycteridocoptes poppei Oudemans, 1898*	3	*Myotis blythii* (Tomes, 1857) and *M. blythii oxygnathus* Monticelli, 1885 [113]
		*Myotis daubentonii* (Leisler *in* Kuhl, 1817) [113]
		*Myotis myotis* (Borkhausen, 1797) [24]
*Oudemansidium musca* (Oudemans, 1906)	1	*Myotis daubentonii* (Leisler *in* Kuhl, 1817) [122, 167, 177]
*Paraperiglischrus rhinolophinus* (C.L. Koch) = *Paraperiglischrus rhinolophinus* (C.L. Koch, 1841)		
			*Paraperiglischrus rhinolophinus* Koch, 1841 = *Paraperiglischrus rhinolophinus* (C.L. Koch, 1841)		
*Paraperiglischrus rhinolophinus* (C.L. Koch, 1841)	3	*Rhinolophus euryale* Blasius, 1853 [50, 76, 138, 167, 235]			
		*Rhinolophus ferrumequinum* (Schreber, 1774) [50, 78, 167, 235]
		*Rhinolophus hipposideros* (Bechstein, 1800) [50, 138, 167, 223?, 235]
*Periglischrus interruptus* (Kolenati, 1856) = *Paraperiglischrus rhinolophinus* (C.L. Koch, 1841)		
*Pteracarus pipistrellius maximis* Uchikawa, 1989	1	*Nyctalus lasiopterus* (Schreber, 1780) [251]
*Prosopodectes chiropteralis* (Trouessart, 1896) = *Notoedres (Notoedres) chiropteralis* (Trouessart, 1896)		
*Psorergates rhinolophi* Fain, 1959	1	*Rhinolophus ferrumequinum* (Schreber, 1774) [108, 167]
*Riedlinia (Riedlinia) petarberoni* (Kolebinova & Vercammen-Grandjean, 1970)	1	*Rhinolophus euryale* Blasius, 1853 [7, 51, 161, 167]
*Sarconissus vespertilionis = Ixodes (Eschatocephalus) vespertilionis* C.L. Koch, 1844		
			*Sarcoptes chiropteralis* Trouessart, 1896 = *Notoedres (Notoedres) chiropteralis* (Trouessart, 1896)		
*Sasatrombicula (Sasatrombicula) bureschi* Kolebinova & Beron, 1965	1	*Rhinolophus hipposideros* (Bechstein, 1800) [51, 52, 162, 167]			
*Sasatrombicula* (*Sasatrombicula*) *hexasternalae* (Vercammen-Grandjean, 1963)	4	Chiroptera gen. sp. [7]
		*Rhinolophus euryale* Blasius, 1853 [51, 162, 167]
		*Rhinolophus ferrumequinum* (Schreber, 1774) [51, 162, 167]
		*Rhinolophus hipposideros* (Bechstein, 1800) [51, 162, 167]
*Spinturnix acuminatus* (C.L. Koch, 1836)	1	*Nyctalus noctula* (Schreber, 1774) [recorded at Col de Bretolet, at the border between France and Switzerland] [79]
*Spinturnix andegavinus* (Deunff, 1977)	1	*Myotis daubentonii* (Leisler *in* Kuhl, 1817) [76], see also [167]

This species is treated as *Spinturnix andegavina* Deunff, 1977 by some authors [167, 209, 225]. According to Sachanowicz *et al.* (publication n°223; p. 49), this species “is a member of the *myoti* species group, which is actually recognised as also containing”: *S. dasycnemi* (Kolenati, 1856)*, S. myoti* (Kolenati, 1856), *S. bechsteini* Deunff *et al.* 2004, *S. emarginata* (Kolenati, 1856), and *S. mystacina* (Kolenati, 1857)”. According to Lanza [167], this taxon is actually a synonym of *Spinturnix daubentonii* (Kolenati, 1857).		
*Spinturnix bechsteini* (Deunff, Walter, Bellido et Volleth, 2004)	1	*Myotis bechsteinii* (Leisler *in* Kuhl, 1817) [82, 130]
*Spinturnix daubentonii* (Kolenati, 1857). This species is treated as a synonym of *Spinturnix andegavinus* (Deunff, 1977) by some authors [167, 209, 225].		
			*Spinturnix helvetiae* (Deunff, Aellen & Keller, 1986).	1	*Nyctalus noctula* (Schreber, 1774) [recorded at Col de Bretolet, at the border between France and Switzerland] [79]
In the opinion of Uchikawa *et al.* (1994), this taxon is actually a subspecies of *S. acuminata* (C.L. Koch, 1836). See the article entitled “Contribution to the taxonomy of the genus *Spinturnix* (Acari: Spinturnicidae), with the erection of a new genus, *Emballonuria*” (*Folia Parasitologica,* 41 (4), p. 295).					
*Spinturnix emarginatus* (Kolenati, 1856)	1	*Myotis emarginatus* (Geoffroy, 1806) [7, 76, 78]

		See also [77]
*Spinturnix euryalis* (G. Canestrini) = *Eyndhovenia euryalis* (G. Canestrini, 1885)		
*Spinturnix murinus* (Walckenaer, 1847) = *Spinturnix myoti* (Kolenati, 1856)		
			*Spinturnix myoti (Kolenati, 1856)*	8	Chiroptera gen. sp. [7, 65?, 167, 246]
*Miniopterus schreibersii* (Natterer *in* Kuhl, 1817) [76]				
*Myotis blythii* (Tomes, 1857) and *M. blythii oxygnathus* Monticelli, 1885 [14, 76]				
*Myotis capaccinii* (Bonaparte, 1837) [76]				
*Myotis myotis* (Borkhausen, 1797) [14?, 76, 78, 177, 226]				
*Myotis nattereri* (Kuhl, 1817) [63, 121]				
*Myotis punicus* Felten, Spitzenberger & Storch, 1977 [64]				
*Rhinolophus euryale* Blasius, 1853 [76]				

See also [77]				
*Spinturnix mystacinus* (Kolenati, 1857)	1	*Myotis mystacinus* (Leisler *in* Kuhl, 1817) [76, 78]

		See also [77]
*Spinturnix nobleti* Deunff, Volleth, Keller et Aellen, 1990	1	*Hypsugo savii* (Bonaparte, 1837) [81, 167]
*Spinturnix oudemansi* van Eyndhoven, 1941 = *Eyndhovenia euryalis* (G. Canestrini, 1885)		
See also *Eyndhovenia euryalis oudemansi* (Eyndhoven, 1941).		

*Spinturnix plecotina* (C.L. Koch, 1839)	3	Chiroptera gen. sp. [7]
		*Plecotus auritus* (Linné, 1758) [63, 76, 167]
		*Plecotus austriacus* Fischer, 1829 [63, 76, 167]
*Spinturnix plecotinus* Koch, 1839 = *Spinturnix plecotina* (C.L. Koch, 1839)		
			*Spinturnix punctata* (Sundevall, 1833)	1	*Barbastella barbastellus* (Schreber, 1774) [80, 167]
*Spinturnix psi* (Kolenati, 1856)	7	Chiroptera gen. sp. [7, 167, 210?, 223]			
		*Eptesicus serotinus* (Schreber, 1774) [48]
		*Miniopterus schreibersii* (Natterer *in* Kuhl, 1817) [50, 63, 76, 139]
		*Myotis capaccinii* (Bonaparte, 1837) [63, 76, 121, 139]
		*Myotis myotis* (Borkhausen, 1797) [76]
		*Rhinolophus euryale* Blasius, 1853 [76]
		*Rhinolophus ferrumequinum* (Schreber, 1774) [139, 223?]

		See also [210]
*Spinturnix* sp.	1	*Myotis myotis* (Borkhausen, 1797) [133]
*Steatonyssus* sp.	1	*Myotis myotis* (Borkhausen, 1797) [152, 212]
*Steatonyssus spinosus* Willmann, 1936	1	*Rhinolophus ferrumequinum* (Schreber, 1774) [50, 51, 167]
*Thrombicula autumnalis* Shaw = *Neotrombicula autumnalis* (Shaw, 1790)		
			*Thrombicula russica* Oudemans = *Leptotrombidium russicum* (Oudemans, 1902)		
*Thrombicula russicum* = *Leptotrombidium russicum* (Oudemans, 1902)					
			*Trombicula muscae* = *Oudemansidium musca* (Oudemans, 1906)		
*Trombicula russicum* = *Leptotrombidium russicum* (Oudemans, 1902)					

**Acari, part 2/3: invalid taxa (*n* = 22)**		
[*Acarus vespertilionis* Hermann]	1	Chiroptera gen. sp. [257, 258]
[*Acarus vespertilionis* Hermann, 1804 – la Mite de la chauve-souris]	1	*Nyctalus noctula* (Schreber, 1774)? [90, 135]
[*Argas caris*]	1	*Pipistrellus pipistrellus* (Schreber, 1774) [191]
[*Caris vespertilionis* = *Argas pipistrellae* (Audouin)? = *Argas vespertilionis* (Latreille, 1796)?]	2?	*Nyctalus noctula* (Schreber, 1774)? [168?, 171, 201]
		*Pipistrellus pipistrellus* (Schreber, 1774)? [258]
		See also [90]
[*Dermanyssus coriaceus* Gervais (=*Hirstionyssus arcuatus* (Koch, 1839)?)]	1	*Nyctalus noctula* (Schreber, 1774) [11, 13, 103, 212, 222, 258, 259]
[*Dermanyssus murinus* (Lucas, 1840)]	1	*Myotis myotis* (Borkhausen, 1797) [101, 104, 167, 183, 195]
[*Dermanyssus vespertilionis* Dugès, 1834]	1	Chiroptera gen. sp. [94]
[*Gamassus dermanyssoïdes* (Mégnin)]	1	*Myotis mystacinus* (Leisler *in* Kuhl, 1817) [248]
[*Gamasus pteroptoides* (Mégnin)]	1	*Pipistrellus pipistrellus* (Schreber, 1774) [190]
The species is recognised by Da Fonseca [72], Lanza [167] and Neumann (see *A treatise on the parasites and parasitic diseases of the domesticated animals.* Translation George Fleming, 2nd edition. Baillière Tindall, London, 1892, 574 p.; p. 99). Kramer treated the species as a synonym of *Laelaps pteroptoides* Mégnin (see the article entitled ‘Ueber Milben’, published in *Archiv für Naturgeschichte,* volume 52, issue 1, 1886, p. 251). Tiraboschi [244] treated the species as a synonym of *Haemomyson musculi* and *Laelaps (Iphis) agilis* Koch, Berlese. According to Rudnick [223], *Gamasus pteroptoides* (Mégnin) is not a species of the Spinturnicidae family.		
[*Haemogamasus setosus* (Kolenati)]	1	*Rhinolophus ferrumequinum* (Schreber, 1774) [5]
[*Haemomyson trouessarti* (Mégnin) (=*Leiognathus arcuatus*=*Hirstionyssus arcuatus* (Koch, 1839)? (See [167, 244], p. 119)	3	*Pipistrellus pipistrellus* (Schreber, 1774) [191, 244]
		*Nyctalus noctula* (Schreber, 1774) [191, 244]
		[*Vesperugo nystagmus*] [191, 244]. This synonym is not mentioned by Didier and Rode in *Les Mammifères de France*
[*Laelaps (Iphis) agilis* Koch, Berlese = *Gamasus pteroptoides* (Mégnin)? = *Laelaps agilis* C.L. Koch, 1836?]	1	*Pipistrellus pipistrellus* (Schreber, 1774) ([244], see also [190])
			[*Leiognathus armatus (=Hirstionyssus arcuatus* (C.L. Koch, 1839)?)]	1	Chiroptera gen. sp. [160, 177]
[*Liponyssus arcuatus* Koch (=*Hirstionyssus arcuatus* (C.L. Koch, 1839) (*pro parte*) and *Steatonyssus murinus* (Lucas, 1840) (*pro parte*)]	1	*Rhinolophus ferrumequinum* (Schreber, 1774) [200]
			[*Liponyssus decussatus* (Kolenati) (=*Caris decussata* Kolenati, 1856?)]	1	*Myotis myotis* (Borkhausen, 1797) [23]
[*Liponyssus setosus* (Kolenati)]	2?	Chiroptera gen. sp. [72]
		*Rhinolophus* sp. [72]
[*Pteroptus trouessarti* Mégnin (=*Haemomyson trouessarti* (Mégnin)?)]	2	*Myotis daubentonii* (Leisler *in* Kuhl, 1817) [177, 248]
		*Myotis mystacinus* (Leisler *in* Kuhl, 1817) [177, 248]
[*Pteroptus vespertilionis*]	3	Absence in *Pipistrellus pipistrellus* (Schreber, 1774) [67]

		*Barbastella barbastellus* (Schreber, 1774) [67]
		Chiroptera gen. sp. [160]
		*Nyctalus noctula* (Schreber, 1774) [131]
[*Pteroptus vespertilionis* Hermann]	2	*Rhinolophus ferrumequinum* (Schreber, 1774) [215]
		*Pipistrellus pipistrellus* (Schreber, 1774) [215]
[*Pteroptus vespertilionis* (Dufour, 1832)]	9	*Barbastella barbastellus* (Schreber, 1774) [127, 177]
		Chiroptera gen. sp. [120, 127]
		*Eptesicus serotinus* (Schreber, 1774) [190]
		*Miniopterus schreibersii* (Natterer *in* Kuhl, 1817) [186]
		*Myotis myotis* (Borkhausen, 1797) [90?, 92]
		*Myotis mystacinus* (Leisler *in* Kuhl, 1817) [248]
		*Nyctalus noctula* (Schreber, 1774) [131?, 177]
		*Rhinolophus ferrumequinum* (Schreber, 1774) [200]
		*Rhinolophus hipposideros* (Bechstein, 1800) [200]
		See also [134, 193, 235]
[*Spinturnix vespertilionis*]	2	*Miniopterus schreibersii* (Natterer *in* Kuhl, 1817) [24]
		*Myotis myotis* (Borkhausen, 1797) [25]
[*Spinturnix vespertilionis* (C.L. Koch)]	3	*Miniopterus schreibersii* (Natterer *in* Kuhl, 1817) [70]
		*Myotis myotis* (Borkhausen, 1797) [70]
		*Plecotus auritus* (Linné, 1758) [70]


**Acari, part 3/3: taxa only noted as absent (*n* = 3)**		
*Ixodes canisuga* Johnston, 1849		Absence in *Barbastella barbastellus* (Schreber, 1774) [28]
		Absence in *Eptesicus serotinus* (Schreber, 1774) [28]
		Absence in *Myotis bechsteinii* (Leisler *in* Kuhl, 1817) [28]
		Absence in *Myotis daubentonii* (Leisler *in* Kuhl, 1817) [28]
		Absence in *Myotis emarginatus* (Geoffroy, 1806) [28]
		Absence in *Myotis myotis* (Borkhausen, 1797) [28]
		Absence in *Myotis mystacinus* (Leisler *in* Kuhl, 1817) [28]
		Absence in *Myotis nattereri* (Kuhl, 1817) [28]
		Absence in *Pipistrellus kuhlii* (Kuhl, 1817) [28]
		Absence in *Pipistrellus nathusii* (Keyserling & Blasius, 1839) [28]
		Absence in *Pipistrellus pipistrellus* (Schreber, 1774) [28]
		Absence in *Plecotus auritus* (Linné, 1758) [28]
		Absence in *Rhinolophus euryale* Blasius, 1853 [28]
		Absence in *Rhinolophus ferrumequinum* (Schreber, 1774) [28]
		Absence in *Rhinolophus hipposideros* (Bechstein, 1800) [28]

[*Ixodes ricinus* (Linné, 1746)]		Absence in *Barbastella barbastellus* (Schreber, 1774) [28]
		Absence in *Eptesicus serotinus* (Schreber, 1774) [28]
		Absence in *Myotis bechsteinii* (Leisler *in* Kuhl, 1817) [28]
		Absence in *Myotis daubentonii* (Leisler *in* Kuhl, 1817) [28]
		Absence in *Myotis emarginatus* (Geoffroy, 1806) [28]
		Absence in *Myotis myotis* (Borkhausen, 1797) [28]
		Absence in *Myotis mystacinus* (Leisler *in* Kuhl, 1817) [28]
		Absence in *Myotis nattereri* (Kuhl, 1817) [28]
		Absence in *Pipistrellus kuhlii* (Kuhl, 1817) [28]
		Absence in *Pipistrellus nathusii* (Keyserling & Blasius, 1839) [28]
		Absence in *Pipistrellus pipistrellus* (Schreber, 1774) [28]
		Absence in *Plecotus auritus* (Linné, 1758) [28]
		Absence in *Rhinolophus euryale* Blasius, 1853 [28]
		Absence in *Rhinolophus ferrumequinum* (Schreber, 1774) [28]
		Absence in *Rhinolophus hipposideros* (Bechstein, 1800) [28]

*Pholeoixodes hexagonus* (Leach, 1815)		Absence in *Barbastella barbastellus* (Schreber, 1774) [28]
		Absence in *Eptesicus serotinus* (Schreber, 1774) [28]
		Absence in *Myotis bechsteinii* (Leisler *in* Kuhl, 1817) [28]
		Absence in *Myotis daubentonii* (Leisler *in* Kuhl, 1817) [28]
		Absence in *Myotis emarginatus* (Geoffroy, 1806) [28]
		Absence in *Myotis myotis* (Borkhausen, 1797) [28]
		Absence in *Myotis mystacinus* (Leisler *in* Kuhl, 1817) [28]
		Absence in *Myotis nattereri* (Kuhl, 1817) [28]
		Absence in *Pipistrellus kuhlii* (Kuhl, 1817) [28]
		Absence in *Pipistrellus nathusii* (Keyserling & Blasius, 1839) [28]
		Absence in *Pipistrellus pipistrellus* (Schreber, 1774) [28]
		Absence in *Plecotus auritus* (Linné, 1758) [28]
		Absence in *Rhinolophus euryale* Blasius, 1853 [28]
		Absence in *Rhinolophus ferrumequinum* (Schreber, 1774) [28]
		Absence in *Rhinolophus hipposideros* (Bechstein, 1800) [28]

**Siphonaptera (*n* = 12)**		
*Araeopsylla gestroi* (Rothschild, 1906)	1	*Tadarida teniotis* (Rafinesque, 1814) [2, 37, 44, 167]
*Ceratopsyllus hexactenus* = *Ischnopsyllus (Hexactenopsylla) hexactenus* (Kolenati, 1856)		
			*Ceratopsyllus octactenus = Ischnopsyllus (Ischnopsyllus) octactenus* (Kolenati, 1856)		
*Ceratopsyllus pentactenus = Nycteridopsylla pentactena* (Kolenati, 1856)					
			*Ceratopsyllus unipectinatus* Taschenberg = *Rhinolophopsylla unipectinata unipectinata* (Taschenberg, 1880)		
*Ischnopsyllus (Ischnopsyllus) elongatus* (Curtis, 1832)	1	*Nyctalus noctula* (Schreber, 1774) [37, 42, 44, 167, 204, 228!, 231!]			
*Ischnopsyllus (Hexactenopsylla) hexactenus* (Kolenati) = *Ischnopsyllus (Hexactenopsylla) hexactenus* (Kolenati, 1856)		
			*Ischnopsyllus (Hexactenopsylla) hexactenus* (Kolenati, 1856)	9	*Barbastella barbastellus* (Schreber, 1774) [2, 29, 33, 37, 38, 44, 200]
Chiroptera gen. sp. [142]				
*Myotis blythii* (Tomes, 1857) and *M. blythii oxygnathus* Monticelli, 1885? [14]				
*Myotis myotis* (Borkhausen, 1797) [14?, 29, 33, 37, 167, 228]				
*Myotis* sp. [44]				
*Pipistrellus kuhlii* (Kuhl, 1817) [29]				
*Plecotus auritus* (Linné, 1758) [2, 33, 37, 44, 142, 177, 227, 228]				
*Plecotus austriacus* Fischer, 1829 [33, 37, 44]				
*Rhinolophus ferrumequinum* (Schreber, 1774) [152]				
*Ischnopsyllus (Ischnopsyllus) octactenus* (Kolenati, 1856)	7	*Barbastella barbastellus* (Schreber, 1774) [200, 228]
		Chiroptera gen. sp. [219]
		*Myotis myotis* (Borkhausen, 1797) [25, 29, 37, 167]
		*Pipistrellus kuhlii* (Kuhl, 1817) [29, 33, 37, 167]
		*Pipistrellus nathusii* (Keyserling & Blasius, 1839) [29, 33, 37, 167]
		*Pipistrellus pipistrellus* (Schreber, 1774) [2, 29, 33, 36, 37, 44, 142, 164, 167, 177, 227]
		*Pipistrellus* sp. [142]
*Ischnopsyllus (Ischnopsyllus) intermedius* (Rothschild) = *Ischnopsyllus (Ischnopsyllus) intermedius* (Rothschild, 1898)		
			*Ischnopsyllus (Ischnopsyllus) intermedius* (Rothschild, 1898)	6	Chiroptera gen. sp. [44]
*Eptesicus serotinus* (Schreber, 1774) [29, 33, 37, 38, 142, 167, 227]				
*Myotis myotis* (Borkhausen, 1797) [2, 29, 33, 37, 142, 167]				
*Nyctalus leisleri* (Kuhl, 1817) [44]				
*Nyctalus noctula* (Schreber, 1774) [3, 44]				
*Rhinolophus ferrumequinum* (Schreber, 1774) [29]				
*Ischnopsyllus (Ischnopsyllus) simplex* Rothschild, 1906	8	*Barbastella barbastellus* (Schreber, 1774) [29, 37]
		*Myotis daubentonii* (Leisler *in* Kuhl, 1817) [44]
		*Myotis emarginatus* (Geoffroy, 1806) [29, 37, 142]
		*Myotis myotis* (Borkhausen, 1797) [29, 37, 85?]
		*Myotis mystacinus* (Leisler *in* Kuhl, 1817) [2, 29, 33, 37, 44, 85?, 142, 156]
		*Myotis nattereri* (Kuhl, 1817) [29, 33, 37, 44, 85?, 142]
		*Plecotus auritus* (Linné, 1758)? [29, 37]
		*Rhinolophus ferrumequinum* (Schreber, 1774)? [152, 177]
		See also [167]
*Ischnopsyllus (Ischnopsyllus) variabilis* Wagner = *Ischnopsyllus (Ischnopsyllus) variabilis* (Wagner, 1898)		
			*Ischnopsyllus (Ischnopsyllus) variabilis* (Wagner, 1898)	6	*Myotis daubentonii* (Leisler *in* Kuhl, 1817) [44]
*Nyctalus noctula* (Schreber, 1774) [37]				
*Pipistrellus kuhlii* (Kuhl, 1817) [37, 44, 142]				
*Pipistrellus nathusii* (Keyserling & Blasius, 1839) [2, 29, 33, 37, 44]				
*Pipistrellus pipistrellus* (Schreber, 1774) [33, 37, 85]				
*Plecotus auritus* (Linné, 1758) ! [85]				
*Nycteridopsylla ancyluris ancyluris* Jordan, 1942	1	*Pipistrellus pipistrellus* (Schreber, 1774) [33, 37, 44, 142, 157, 164]
*Nycteridopsylla dictena* (Kolenati, 1856) !	1	Chiroptera gen. sp.! Doubtful : see Beaucournu et Launay (1990 ; p. 314 [paper n°^37^]) [37, 146, 167, 227]
*Nycteridopsylla dictenus* (Kolenati, 1857) = *Nycteridopsylla dictena* (Kolenati, 1856)		
			*Nycteridopsylla (Dinycteropsylla) dictena* (Kolenati, 1856) = *Nycteridopsylla dictena* (Kolenati, 1856)		
*Nycteridopsylla eusarca* Dampf, 1908	1	*Nyctalus noctula* (Schreber, 1774) [37, 42]			
*Nycteridopsylla longiceps* Rothschild, 1908	10	*Barbastella barbastellus* (Schreber, 1774) [29, 33, 37, 167]
		*Myotis capaccinii* (Bonaparte, 1837) [37, 164, 167]
		*Myotis daubentonii* (Leisler *in* Kuhl, 1817) [29, 37, 167]
		*Myotis myotis* (Borkhausen, 1797) ! [228]
		*Myotis mystacinus* (Leisler *in* Kuhl, 1817) [29, 37, 38, 167]
		*Myotis nattereri* (Kuhl, 1817) [29, 37, 167]
		*Pipistrellus kuhlii* (Kuhl, 1817) [37, 44, 156]
		*Pipistrellus nathusii* (Keyserling & Blasius, 1839) [37, 44, 167]
		*Pipistrellus pipistrellus* (Schreber, 1774) [2, 29, 33, 37, 85, 167]
		*Plecotus auritus* (Linné, 1758) [33, 37, 85, 167, 227]
*Nycteridopsylla pentactena* (Kolenati, 1856)	13	*Barbastella barbastellus* (Schreber, 1774) [2, 29, 33, 37, 44, 167, 200]
		Chiroptera gen. sp. [187]
		*Eptesicus serotinus* (Schreber, 1774) [29, 33, 37, 164, 167]
		*Miniopterus schreibersii* (Natterer *in* Kuhl, 1817) [37, 164, 167]
		*Myotis capaccinii* (Bonaparte, 1837) [37, 164, 167]
		*Myotis myotis* (Borkhausen, 1797) [29, 37, 167]
		*Myotis mystacinus* (Leisler *in* Kuhl, 1817) [29, 37, 167]
		*Myotis nattereri* (Kuhl, 1817) [29, 37, 167]
		*Pipistrellus nathusii* (Keyserling & Blasius, 1839) [44]
		*Pipistrellus pipistrellus* (Schreber, 1774) [29, 37, 38, 167]
		*Plecotus auritus* (Linné, 1758) [29, 33, 37, 167]
		*Plecotus austriacus* Fischer, 1829 [33, 37, 167]
		*Rhinolophus ferrumequinum* (Schreber, 1774) [29, 37, 152, 164, 167, 177]
		See also [217]
*Pulex pungens* Walckenaer (1802) (*in* Faune parisienne) = *Nycteridopsylla eusarca* Dampf, 1908		
			[*Pulex vespertilionis* Dugès, 1832]	1	*Myotis myotis* (Borkhausen, 1797) [95]
See also [90]				
*Rhinolophopsylla unipectinata unipectinata* (Taschenberg, 1880)	4	*Rhinolophus euryale* Blasius, 1853 [2, 29, 33, 142, 167]
		*Rhinolophus ferrumequinum* (Schreber, 1774) [2, 33, 38, 44, 142, 152, 156, 164, 167, 177, 227, 242]
		*Rhinolophus hipposideros* (Bechstein, 1800) [2, 86, 142, 167, 200, 228]
		*Rhinolophus* sp. [44]
**Diptera (*n* = 13)**		
*Basilia italica* Theodor = *Basilia (Basilia) italica* Theodor, 1954		
			*Basilia nana* Theodor = *Basilia (Basilia) nana* Theodor & Moscona, 1954		
*Basilia nattereri* (Kolenati) = *Basilia (Basilia) nattereri* (Kolenati, 1857)		
			*Basilia (Basilia) italica* Theodor, 1954	2	*Myotis emarginatus* (Geoffroy, 1806) [4, 34]
*Myotis mystacinus* (Leisler *in* Kuhl, 1817) [34, 35, 43, 237]				
See also [28, 150, 167]				
*Basilia (Basilia) mediterranea* Hůrka, 1970	4	*Eptesicus serotinus* (Schreber, 1774) [43, 154, 237]
		*Hypsugo savii* (Bonaparte, 1837) [41, 167, 237]
		*Pipistrellus pipistrellus* (Schreber, 1774) [40, 43, 167]
		*Pipistrellus* sp. [41]
*Basilia (Basilia) nana* Theodor & Moscona, 1954	5	*Myotis bechsteinii* (Leisler *in* Kuhl, 1817) [4, 28, 30, 38, 237]
		*Myotis emarginatus* (Geoffroy, 1806) [43, 237]
		*Myotis mystacinus* (Leisler *in* Kuhl, 1817) [241]
		*Myotis nattereri* (Kuhl, 1817) [4, 30, 237]
		*Plecotus auritus* (Linné, 1758) [28, 237]
		See also [150, 167]
*Basilia (Basilia) nattereri* (Kolenati, 1857)	4	*Eptesicus serotinus* (Schreber, 1774) [241]
		*Myotis daubentonii* (Leisler *in* Kuhl, 1817) [187]
		*Myotis nattereri* (Kuhl, 1817) [4, 28, 30, 43, 187]
		*Plecotus auritus* (Linné, 1758) [28, 187]
		See also [150, 167, 237]
*Brachytarsina flavipennis* Macquart, 1851	1	*Rhinolophus ferrumequinum* (Schreber, 1774) [26, 40, 43, 117, 155, 237]
*Celeripes biarticulata = Phthiridium biarticulatum* Hermann, 1804		
			*Listropodia pedicularia = Nycteribia (Nycteribia) pedicularia* Latreille, 1805		
*Listropodia Schmidli = Nycteribia (Nycteribia) schmidlii* Schiner, 1853					
			*Nycteribia (Achrocholidia) vexata* Westwood = *Nycteribia (Achrocholidia) vexata* Westwood, 1835		
*Nycteribia (Achrocholidia) vexata* Westwood, 1835	8	*Myotis emarginatus* (Geoffroy, 1806) ! [23]			
		*Rhinolophus euryale* Blasius, 1853 ! [85]
		*Rhinolophus ferrumequinum* (Schreber, 1774) ! [85]
		*Rhinolophus hipposideros* (Bechstein, 1800) ! [85]

		*Miniopterus schreibersii* (Natterer *in* Kuhl, 1817) [1, 85, 167]
		*Myotis blythii* (Tomes, 1857) and *M. blythii oxygnathus* Monticelli, 1885 [30]
		*Myotis myotis* (Borkhausen, 1797) [1, 23, 25, 28, 30, 38, 167]
		*Myotis myotis* (Borkhausen, 1797)/*Miniopterus schreibersii* (Natterer *in* Kuhl, 1817) [43]
		See also [117, 150, 237]
*Nycteribia (Celeripes) biarticulata* Hermann = *Phthiridium biarticulatum* Hermann, 1804		
			*Nycteribia biarticulata = Phthiridium biarticulatum* Hermann, 1804		
*Nycteribia blasii* (Kolenati) = *Nycteribia (Nycteribia) kolenatii* Theodor & Moscona, 1954		
			*Nycteribia Celeripes Bi-Articulata = Phthiridium biarticulatum* Hermann, 1804		
*Nycteribia (Celeripes) biarticulata* Hermann 1804 = *Phthiridium biarticulatum* Hermann, 1804		
*Nycteribia Hermanni* Leach = *Phthiridium biarticulatum* Hermann, 1804		
			*Nycteribia kolenatii* Theodor = *Nycteribia (Nycteribia) kolenatii* Theodor & Moscona, 1954		
*Nycteribia latreilli* Leach = *Nycteribia (Nycteribia) latreillii* (Leach, 1817)					
			*Nycteribia (Listropodia) Schmidti* Schiner = *Nycteribia (Nycteribia) schmidlii* Schiner, 1853		
*Nycteribia pedicularia* = *Nycteribia (Nycteribia) pedicularia* Latreille, 1805)					
			*Nycteribia schmidli* = *Nycteribia (Nycteribia) schmidlii* Schiner, 1853		
*Nycteribia vespertilionis* Dufour, 1831 = *Penicillidia (Penicillidia) dufourii* (Westwood, 1834)					
			*Nycteribia vexata = Nycteribia (Achrocholidia) vexata* Westwood, 1835		
*Nycteribia (Nycteribia) kolenatii* Theodor & Moscona, 1954	6	*Eptesicus serotinus* (Schreber, 1774) [147, 241]			
		*Miniopterus schreibersii* (Natterer *in* Kuhl, 1817) [200]
		*Myotis daubentonii* (Leisler *in* Kuhl, 1817) [1, 28, 38, 43, 167, 241]
		*Myotis mystacinus* (Leisler *in* Kuhl, 1817) [147, 241]
		*Myotis nattereri* (Kuhl, 1817) [28, 147]
		*Pipistrellus pipistrellus* (Schreber, 1774) [241]
		See also [150, 237, 240]
*Nycteribia (Nycteribia) latreillii* (Leach, 1817)	6	Chiroptera gen. sp. [148]
		*Miniopterus schreibersii* (Natterer *in* Kuhl, 1817) [1, 167]
		*Myotis blythii* (Tomes, 1857) and *M. blythii oxygnathus* Monticelli, 1885 [30, 167]
		*Myotis emarginatus* (Geoffroy, 1806) [22]
		*Myotis myotis* (Borkhausen, 1797) [1, 28, 30]
		*Myotis myotis* (Borkhausen, 1797)/*Miniopterus schreibersii* (Natterer *in* Kuhl, 1817) [43]
		See also [147, 150, 237]
*Nycteribia (Nycteribia) pedicularia* Latreille, 1805	12	Chiroptera gen. sp.? [170]
		*Miniopterus schreibersii* (Natterer *in* Kuhl, 1817) ! [85]
		*Myotis daubentonii* (Leisler *in* Kuhl, 1817) ! [85]
		*Myotis dasycneme* (Boie, 1825) ! [85]
		*Myotis emarginatus* (Geoffroy, 1806)? [23]
		*Nyctalus noctula* (Schreber, 1774) ! [85]
		*Pipistrellus pipistrellus* (Schreber, 1774)? [214, 215]
		*Rhinolophus euryale* Blasius, 1853 ! [85]
		*Rhinolophus hipposideros* (Bechstein, 1800)? [200]

		*Myotis blythii* (Tomes, 1857) and *M. blythii oxygnathus* Monticelli, 1885 [115, 154, 167]
		*Myotis capaccinii* (Bonaparte, 1837) [1, 30, 85?]
		*Myotis myotis* (Borkhausen, 1797) [25?, 186]
		See also [28, 85, 117, 149, 150, 177, 189?, 206?, 237]. About old records of *Nycteribia pedicularia* see Szentiványi *et al*., 2016; p. 102 [237]
*Nycteribia (Nycteribia) schmidlii* Schiner, 1853	7	*Miniopterus schreibersii* (Natterer *in* Kuhl, 1817) [1, 24, 28, 30, 43, 115, 149, 154, 167, 242] see also [117, 150, 237]
		*Miniopterus schreibersii* (Natterer *in* Kuhl, 1817)/ *Myotis myotis* (Borkhausen, 1797) [30]
		*Myotis blythii* (Tomes, 1857) and *M. blythii oxygnathus* Monticelli, 1885 [30]
		*Myotis daubentonii* (Leisler *in* Kuhl, 1817) [147]
		*Myotis myotis* (Borkhausen, 1797) [1, 30]
		*Pipistrellus pipistrellus* (Schreber, 1774) [147]
		*Rhinolophus ferrumequinum* (Schreber, 1774) [1]
		*Rhinolophus mehelyi* Matschie, 1901 [147]
*Nycteribia* sp.	3	*Myotis daubentonii* (Leisler *in* Kuhl, 1817) [247]
		*Pipistrellus pipistrellus* (Schreber, 1774) [214, 215]
		*Rhinolophus ferrumequinum* (Schreber, 1774) [73]

		See also [74]
*Nycteribia (Stylidia) biarticulata* Hermann = *Phthiridium biarticulatum* Hermann, 1804		
			*Nycteribia (Stylidia) biarticulata = Phthiridium biarticulatum* Hermann, 1804		
[*Nycteribia eparticulata*]	1	*Myotis daubentonii* (Leisler *in* Kuhl, 1817) [248]			
[*Nycteribia frauenfeldi* (Kolenati)] = *Penicillidia (Penicillidia) dufourii* (Westwood, 1834)		
[*Nycteribia vespertilionis* Latreille]	1	*Rhinolophus ferrumequinum* (Schreber, 1774) [73]
Nyctéribie du Vespertilion	1?	Chiroptera gen. sp.? [89]
[*Nycteribia vespertilionis* Meig.]	2	Chiroptera gen. sp. [12]
		*Miniopterus schreibersii* (Natterer *in* Kuhl, 1817) [203]
*Nycteribosca kollari* Frauenfeld, 1855 = *Brachytarsina flavipennis* Macquart, 1851		
			Complex of species *Nycteribia kolenatii/latreillii/pedicularia*	1	*Pipistrellus pipistrellus* (Schreber, 1774) [177, 214, 215]
*Penicillidia conspicua* Speiser = *Penicillidia (Neopenicillidia) conspicua* Speiser, 1901		
			*Penicillidia d. dufourii* (Westwood, 1834) = *Penicillidia (Penicillidia) dufourii* (Westwood, 1834)		
*Penicillidia dufouri* Westwood = *Penicillidia (Penicillidia) dufourii* (Westwood, 1834)		
			*Penicillidia (Neopenicillidia) conspicua* Speiser, 1901	2	*Miniopterus schreibersii* (Natterer *in* Kuhl, 1817) [1, 28, 30, 115, 154, 167, 242], see also [117, 150, 237]
*Myotis myotis* (Borkhausen, 1797) [25]				
*Penicillidia (Penicillidia) dufourii* (Westwood, 1834)	7	Chiroptera gen. sp. [43]
		*Miniopterus schreibersii* (Natterer *in* Kuhl, 1817) [1, 24, 30, 237]
		*Myotis blythii* (Tomes, 1857) and *M. blythii oxygnathus* Monticelli, 1885 [14?, 43, 115?, 154?, 237]
		*Myotis capaccinii* (Bonaparte, 1837) [1, 237]
		*Myotis myotis* (Borkhausen, 1797) [1, 14?, 22, 28, 30, 43, 90? 91?, 186?, 237, 242?]
		*Pipistrellus* sp. [53]
		*Rhinolophus ferrumequinum* (Schreber, 1774)? [200]
		See also [93?, 117?, 150, 167, 260?]
*Phthiridium biarticulatum* Hermann, 1804	6	Chiroptera gen. sp.? [173?, 204?]
		*Miniopterus schreibersii* (Natterer *in* Kuhl, 1817)? [85, 126, 237]
		*Plecotus auritus* (Linné, 1758) [85, 86, 237]
		*Rhinolophus euryale* Blasius, 1853 [28, 30, 38, 115, 167, 242]
		*Rhinolophus ferrumequinum* (Schreber, 1774) [1, 28, 30, 43, 85, 115, 126?, 135?, 151, 152, 154, 156, 175?, 177, 237, 242]
		*Rhinolophus hipposideros* (Bechstein, 1800) [115, 126?, 135?, 167, 237]
		See also [47, 116, 117, 150]
*Penicillidia (Penicillidia) monoceros* Speiser, 1900		Absence in *Barbastella barbastellus* (Schreber, 1774) [28]
		Absence in *Eptesicus serotinus* (Schreber, 1774) [28]
		Absence in *Myotis bechsteinii* (Leisler *in* Kuhl, 1817) [28]
		Absence in *Myotis daubentonii* (Leisler *in* Kuhl, 1817) [28]
		Absence in *Myotis emarginatus* (Geoffroy, 1806) [28]
		Absence in *Myotis myotis* (Borkhausen, 1797) [28]
		Absence in *Myotis mystacinus* (Leisler *in* Kuhl, 1817) [28]
		Absence in *Myotis nattereri* (Kuhl, 1817) [28]
		Absence in *Pipistrellus kuhlii* (Kuhl, 1817) [28]
		Absence in *Pipistrellus nathusii* (Keyserling & Blasius, 1839) [28]
		Absence in *Pipistrellus pipistrellus* (Schreber, 1774) [28]
		Absence in *Plecotus auritus* (Linné, 1758) [28]
		Absence in *Rhinolophus euryale* Blasius, 1853 [28]
		Absence in *Rhinolophus ferrumequinum* (Schreber, 1774) [28]
		Absence in *Rhinolophus hipposideros* (Bechstein, 1800) [28]
**Hemiptera (*n* = 3)**		
*Cimex dissimilis* (Horváth, 1910)	4 (5?)	Chiroptera gen. sp. [207]
		*Myotis emarginatus* (Geoffroy, 1806) [254]
		*Myotis myotis* (Borkhausen, 1797)? [177]
		*Myotis* sp. [253]
		*Rhinolophus ferrumequinum* (Schreber, 1774) [254]
		See also [167, 236]
*Cimex lectularius* Merrett 1667 = *Cimex lectularius* Linnaeus, 1758		
*Cimex lectularius* Linnaeus, 1758	1 (5?)	Chiroptera gen. sp. [28, 35]
		*Myotis emarginatus* (Geoffroy, 1806)? [253, 254]
		*Myotis myotis* (Borkhausen, 1797)? [253, 254]
		*Rhinolophus euryale* Blasius, 1853? [253, 254]
		*Rhinolophus ferrumequinum* (Schreber, 1774)? [253, 254]
		See also [167, 236]
*Cimex pipistrelli* Jenins, 1839	1	Chiroptera gen. sp. [28, 236]
*Cimex stadleri* Horváth 1935 = *Cimex dissimilis* (Horváth, 1910)		
			*Cimex* sp.	1	Chiroptera gen. sp. [28, 177]
**Anoplura (*n* = 1)**					
*Polyplax serrata* (Burmeister, 1839) !	1 !	*Rhinolophus hipposideros* (Bechstein, 1800) ! [17, 177, 216]
			**Cestoda (*n* = 3)**		
*Hymenolepis acuta = Vampirolepis acuta* (Rudolphi, 1819)		
*Hymenolepis balsaci = Vampirolepis balsaci* (Joyeux & Baer, 1934)		
*Hymenolepis grisea = Milina grisea* van Beneden, 1873		
*Hymenolepis grisea* (P.J. Van Beneden 1873) = *Milina grisea* van Beneden, 1873		
*Hymenolepis sp. = Vampirolepis species*		
*Milina grisea* van Beneden, 1873	4 (5?)	*Miniopterus schreibersii* (Natterer *in* Kuhl, 1817) [68, 86, 167]
		*Myotis emarginatus* (Geoffroy, 1806) [68, 86, 167]
		*Myotis myotis* (Borkhausen, 1797) [68, 159, 167]
		*Rhinolophus ferrumequinum* (Schreber, 1774) [68, 71, 159, 196, 243]
		*Rhinolophus ferrumequinum* or *R. hipposideros* or *M. schreibersii* [200]
*Myotolepis grisea* (Beneden, 1873) = *Milina grisea* van Beneden, 1873		
			*Vampirolepis acuta* (Rudolphi, 1819)	1	*Eptesicus serotinus* (Schreber, 1774) [158, 159, 177, 196, 255]
*Vampirolepis balsaci* (Joyeux & Baer, 1934)	2	*Eptesicus serotinus* (Schreber, 1774) [158, 159, 167, 177, 180, 196, 255]
		*Myotis bechsteinii* (Leisler *in* Kuhl, 1817) [159, 167, 177, 180, 196, 255]
*Vampirolepis sp. / Milina sp.*	2	*Eptesicus serotinus* (Schreber, 1774) [86]
		*Plecotus auritus* (Linné, 1758) [86]
**Trematoda (*n* = 15)**		
*Allassogonoporus amphoraeformis* (Mödlinger, 1930)	1	*Myotis daubentonii* (Leisler *in* Kuhl, 1817) [89, 167]
*Distoma ascidoïdes* Van Beneden, 1873 = *Prosthodendrium (Prosthodendrium) chilostomum* (Mehlis, 1831)		
			*Distoma heteroptum* (Dujardin, 1845) = *Pycnoporus heteroporus* (Dujardin, 1845)		
*Distoma lagena* Brandes, 1888 = *Lecithodendrium granulosum* Looss, 1907					
			*Lecithodendrium granulosum* Looss, 1907	1 (4?)	*Miniopterus schreibersii* (Natterer *in* Kuhl, 1817)? [200]
*Myotis capaccinii* (Bonaparte, 1837) [68]				
*Rhinolophus ferrumequinum* (Schreber, 1774)? [200]				
*Rhinolophus hipposideros* (Bechstein, 1800)? [200]				
*Lecithodendrium linstowi* R.Ph Dollfus 1931 = *Lecithodendrium linstowi* Dollfus, 1931		
			*Lecithodendrium linstowi* Dollfus, 1931	10	*Eptesicus serotinus* (Schreber, 1774) [68, 88, 167]
*Miniopterus schreibersii* (Natterer *in* Kuhl, 1817) [68, 167]				
*Myotis capaccinii* (Bonaparte, 1837) [68, 167]				
*Myotis emarginatus* (Geoffroy, 1806) [68, 167]				
*Myotis myotis* (Borkhausen, 1797) [68, 167]				
*Pipistrellus kuhlii* (Kuhl, 1817) [86, 167]				
*Pipistrellus pipistrellus* (Schreber, 1774) [68, 167]				
*Plecotus austriacus* Fischer, 1829 [68, 167]				
*Rhinolophus euryale* Blasius, 1853 [68, 167]				
*Rhinolophus ferrumequinum* (Schreber, 1774) [68, 167, 243]				
*Lecithodendrium modlingeri* (Pande, 1935) = *Lecithodendrium moedlingeri* (Pande, 1935)		
			*Lecithodendrium moedlingeri* (Pande, 1935)	1	*Rhinolophus ferrumequinum* (Schreber, 1774) [243]
*Lecithodendrium* sp.	1	*Rhinolophus hipposideros* (Bechstein, 1800) [86]
*Lepoderma vespertilionis* (Müller) = *Plagiorchis vespertilionis* (O.F. Müller, 1784)		
			*Limatulum duboisi* Hurkova 1961 = *Parabascus duboisi* (Hurkova, 1961)		
*Mesotretes peregrinus* (Braun, 1900)	3	*Miniopterus schreibersii* (Natterer *in* Kuhl, 1817) [68, 167, 188]			
		*Rhinolophus ferrumequinum* (Schreber, 1774) [68, 71, 88, 167, 188, 243]
		*Rhinolophus hipposideros* (Bechstein, 1800) [68, 167, 188]
		See also [202]
*Parabascus duboisi* (Hurkova, 1961)	1	*Myotis daubentonii* (Leisler *in* Kuhl, 1817) [89, 167]
*Parabascus lepidotus* Looss, 1907	4	*Miniopterus schreibersii* (Natterer *in* Kuhl, 1817) [68, 167]
		*Plecotus austriacus* Fischer, 1829 [68, 167]
		*Rhinolophus euryale* Blasius, 1853 [68, 167]
		*Rhinolophus ferrumequinum* (Schreber, 1774) [68, 167]
*Parabascus semisquamosus* (Braun, 1900)	1	*Pipistrellus pipistrellus* (Schreber, 1774) [60, 68, 167]
[*Paralecithodendrium chilostomum* (Mehlis) (=*Prosthodendrium (Prosthodendrium) chilostomum* (Mehlis, 1831)?)]	3	*Miniopterus schreibersii* (Natterer *in* Kuhl, 1817) [200]
		*Rhinolophus ferrumequinum* (Schreber, 1774) [200]
		*Rhinolophus hipposideros* (Bechstein, 1800)? [200]
*Plagiorchis vespertilionis* (O.F. Müller, 1784)	11	*Eptesicus serotinus* (Schreber, 1774) [68, 167]
		*Miniopterus schreibersii* (Natterer *in* Kuhl, 1817) [68, 167, 200, 235]
		*Myotis capaccinii* (Bonaparte, 1837) [68, 167]
		*Myotis daubentonii* (Leisler *in* Kuhl, 1817) [89, 167]
		*Myotis emarginatus* (Geoffroy, 1806) [68, 86, 167]
		*Myotis myotis* (Borkhausen, 1797) [86]
		*Pipistrellus pipistrellus* (Schreber, 1774) [68, 86, 167]
		*Plecotus auritus* (Linné, 1758) [86, 167]
		*Rhinolophus euryale* Blasius, 1853 [68, 167]
		*Rhinolophus ferrumequinum* (Schreber, 1774) [68, 71, 86–88, 167, 200, 243]
		*Rhinolophus hipposideros* (Bechstein, 1800) [68, 86, 167, 200]
*Prosthodendrium carolinum* J. Hurkova, 1959 = *Prosthodendrium (Prosthodendrium) carolinum* Hurková, 1959		
*Prosthodendrium chilostoma* (Mehlis, 1831) = *Prosthodendrium (Prosthodendrium) chilostomum* (Mehlis, 1831)		
*Prosthodendrium chilostomum* (Mehlis) = *Prosthodendrium (Prosthodendrium) chilostomum* (Mehlis, 1831)		
*Prosthodendrium longiforme* (Bhalerao, 1926) = *Prosthodendrium (Prosthodendrium) longiforme* (Bhalerao, 1926)		
			*Prosthodendrium (Prosthodendrium) carolinum* Hurková, 1959	1	*Rhinolophus ferrumequinum* (Schreber, 1774) [167, 243]
*Prosthodendrium (Prosthodendrium) chilostomum* (Mehlis, 1831)	4	*Miniopterus schreibersii* (Natterer *in* Kuhl, 1817) [68, 167, 200, 235]
		*Myotis myotis* (Borkhausen, 1797) [57, 61, 167]
		*Rhinolophus ferrumequinum* (Schreber, 1774) [200, 243]
		*Rhinolophus hipposideros* (Bechstein, 1800) [86, 167, 200, 235]
*Prosthodendrium (Prosthodendrium) hurkovaae* Dubois, 1960	1	*Myotis daubentonii* (Leisler *in* Kuhl, 1817) [89]
*Prosthodendrium (Prosthodendrium) longiforme* (Bhalerao, 1926)	2	*Eptesicus serotinus* (Schreber, 1774) [88, 167]
		*Rhinolophus ferrumequinum* (Schreber, 1774) [167, 243]
*Prosthodendrium parvouterus* (Bhalerao, 1926)	1	*Miniopterus schreibersii* (Natterer *in* Kuhl, 1817) [68, 167]
*Prosthodendrium* sp.	1	*Plecotus auritus* (Linné, 1758) [86]
*Pycnoporus heteroporus* (Dujardin, 1845)	2	*Pipistrellus kuhlii* (Kuhl, 1817) [86]
		*Pipistrellus pipistrellus* (Schreber, 1774) [57, 61, 68, 97, 163, 167, 235]
**Nematoda (*n* = 13)**		
*Histiostrongylus tipula* (van Beneden, 1873) = *Molinostrongylus tipula* (Beneden, 1873)		
			*Litomosa beaucournui* Bain, 1967 = *Litomosa ottavianii* Lagrange & Bettini, 1948		
*Litomosa desportesi* Bain, 1967 = *Litomosa dogieli* Bogdanov & Vladimirov, 1956					
			*Litomosa dogieli* Bogdanov & Vladimirov, 1956	2	*Myotis emarginatus* (Geoffroy, 1806) [19]
*Myotis myotis* (Borkhausen, 1797) [19]				
*Litomosa filaria* P.J. Van Beneden 1873 = *Litomosa filaria* (Beneden, 1873)		
			*Litomosa filaria* (van Beneden) = *Litomosa filaria* (Beneden, 1873)		
*Litomosa filaria* (Beneden, 1873)	3	*Myotis emarginatus* (Geoffroy, 1806) [86, 167]			
		*Myotis myotis* (Borkhausen, 1797) [75, 86, 167]
		*Plecotus auritus* (Linné, 1758) [19, 167]
*Litomosa ottavianii* Lagrange & Bettini, 1948	5	*Miniopterus schreibersii* (Natterer *in* Kuhl, 1817) [19, 167]
		*Myotis emarginatus* (Geoffroy, 1806) [68, 167]
		*Rhinolophus euryale* Blasius, 1853 [68, 167]
		*Rhinolophus ferrumequinum* (Schreber, 1774) [19, 167]
		*Rhinolophus* sp. [19, 167]
*Molinostrongylus alatus* (Ortlepp, 1932)?	1?	Chiroptera gen. sp.? [167]
*Molinostrongylus tipula* (Beneden, 1873)	2	*Miniopterus schreibersii* (Natterer *in* Kuhl, 1817) [200]
		*Myotis myotis* (Borkhausen, 1797) [56]
*Molinostrongylus ornatus* (Monnig, 1927)	1	*Myotis myotis* (Borkhausen, 1797) [68]
*Molinostrongylus panousei* Dollfus, 1954	1	*Miniopterus schreibersii* (Natterer *in* Kuhl, 1817) [99, 167]
*Ophiostoma mucronatum* (Rudolphi, 1809) = *Seuratum mucronatum* (Rudolphi, 1809)		
			*Pterygodermatites (Neopaucipectines) bovieri* (Blanchard, 1886)	1	*Myotis myotis* (Borkhausen, 1797) [56, 167, 177, 211, 233, 235, 245]
*Rictularia bovieri = Pterygodermatites (Neopaucipectines) bovieri* (Blanchard, 1886)		
			*Rictularia plagiostoma* (Wedl, 1861)!	1!	*Myotis myotis* (Borkhausen, 1797) ! [233]
*Rictularia* sp.	1	*Myotis emarginatus* (Geoffroy, 1806) [86]
*Riouxgolvania nyctali* (Bain & Chabaud, 1979)	1	*Myotis blythii* (Tomes, 1857) and *M. blythii oxygnathus* Monticelli, 1885 [21, 167]
*Riouxgolvania rhinolophi* (Bain & Chabaud, 1968)	2	*Miniopterus schreibersii* (Natterer *in* Kuhl, 1817) [21, 167]
		*Rhinolophus euryale* Blasius, 1853 [20, 167]
*Riouxgolvania* sp.	1	*Myotis blythii* (Tomes, 1857) and *M. blythii oxygnathus* Monticelli, 1885 [86]
*Seuratum mucronatum* (Rudolphi, 1809)	2	*Plecotus auritus* (Linné, 1758) [54, 86, 194]
		*Plecotus* sp. [97]
*Strongylacantha glycirrhyza* van Beneden, 1873	2	*Miniopterus schreibersii* (Natterer *in* Kuhl, 1817) [99, 167, 200]
		*Rhinolophus ferrumequinum* (Schreber, 1774) [68, 167, 200, 243]
*Strongylus tipula* P.J. Van Beneden, 1873 = *Molinostrongylus tipula* (Beneden, 1873)		
			*Trichosomum speciosum* van Beneden, 1873	3	*Miniopterus schreibersii* (Natterer *in* Kuhl, 1817) [200]
*Rhinolophus ferrumequinum* (Schreber, 1774) [200]				
*Rhinolophus hipposideros* (Bechstein, 1800) [200]				
*Uncinaria glycirrhiza* (van Beneden, 1873) = *Strongylacantha glycirrhyza* van Beneden, 1873		

#### 1.2 Subphylum Hexapoda Latreille, 1825

##### 1.2.1 Suborder Anoplura Leach, 1815 (order Phthiraptera Haeckel, 1896)

Only one species has been reported as a bat parasite in France, *i.e. Polyplax serrata*. The scientist Paul Rémy (1894–1962) published in 1948 the only record; this was from *R. hipposideros* (Borkhausen, 1797) in north-eastern France at Trémont-sur-Saulx (Meuse area [177, 216]). The mentioned locality in the original paper, “Frémont-sur-Saulx” [216], contains a typographical error. These field data, dated 1925–1926, and published in the journal La Feuille des Naturalistes, are surprising and dubious [Beaucournu, *in litt.*]. In fact, this species is more likely to be an ectoparasite of mammals of the Rodentia order (e.g. *Apodemus*, *Clethrionomys* and *Mus* genera) and Eulipotyphla (*Crocidura leucodon*) [98, 234]. It should be noted, however, that *Polyplax* sp. was also reported on *Rhinolophus mehelyi* by Gadžiev *et al*. in 1990 in Eastern Europe. These are the only data on *Polyplax* sp. in Lanza’s analysis [167] and hence they may be unreliable. Rémy’s observation is not mentioned in the works of Durden & Musser [98], Ferris [119] and Hopkins [140].

##### 1.2.2 Order Diptera Linnaeus, 1758

According to Szentiványi *et al.*, 17 species of bat flies are currently known in Europe [237]. Thirteen species of bat flies have been reported from France and two more records without identification to species level have been found. These are *Basilia (Basilia) italica*, *B. (Basilia) mediterranea*, *B. (Basilia) nana*, 1954, *B. (Basilia) nattereri, Brachytarsina flavipennis*, *Nycteribia (Achrocholidia) vexata*, *N. (Nycteribia) kolenatii*, *N. (Nycteribia) latreillii, N. (Nycteribia) pedicularia, N. (Nycteribia) schmidlii*, *Penicillidia (Neopenicillidia) conspicua*, *P. (Penicillidia) dufourii, Phthiridium biarticulatum*, *Nycteribia* sp. and *Nycteribia kolenatii/latreillii/pedicularia* [1, 4, 12, 22–30, 34, 35, 38–41, 43, 45, 47, 53, 73, 74, 86, 91, 93, 115–117, 126, 135, 146–152, 154, 156, 167, 170, 172, 173, 175, 177, 179, 186, 187, 200, 203, 204, 206, 214, 215, 237, 240–242, 247, 248, 260]. Three invalid taxa reported from France were found in the analysed papers: *Nycteribia eparticulata*, *N. vespertilionis* Meig., and *N. vespertilionis* Latreille. *Penicillidia (Penicillidia) monoceros* is noted as absent in western France [28]. Another species, *B. (Basilia) daganiae* could be distributed in France [28, 34]. As far as hosts of the bat flies (Diptera: Nycteribiidae and Streblidae) are concerned, *E. serotinus*, *H. savii*, *M. schreibersii*, *M. bechsteinii*, *M. blythii, M. capaccinii, M. daubentonii*, *M. emarginatus*, *M. myotis*, *M. mystacinus*, *M. nattereri*, *M. species, P. pipistrellus*, *Pipistrellus* sp., *P. auritus*, *R. euryale, R. ferrumequinum*, *R. hipposideros*, and *R. mehelyi* have been recorded in the literature. Jean-Frédéric Hermann’s record of *Phthiridium vespertilionis*, dated 1804, is the oldest French record of a dipteran as a bat parasite [135]. The bibliographical survey of the published data (*n* = 111 papers) written by Szentiványi *et al.* has shown that ten bat fly species are known to be associated with bats in Albania, Romania, and Italy [237]. Europe’s most species-rich communities have been reported in Spain (11 species), Switzerland (11 species), Hungary (11 species) and France (13 species). As such, France has the most diverse community reported in the literature.

##### 1.2.3 Order Hemiptera Linnaeus, 1758

The literature provides well-documented cases for *Cimex dissimilis*, *C. lectularius*, and *C. pipistrelli* on *M. myotis, M. emarginatus, R. euryale*, and *R. ferrumequinum* [28, 167, 177, 207, 236, 253, 254]. In addition to these species, records reported from France without identification to species level have been found (*Cimex* sp. on *M. myotis, M. emarginatus, R. euryale*, and *R. ferrumequinum*) [28, 177]. The first published data in the analysed literature are dated 1961 [28] when Beaucournu published his observations on *Cimex* sp. in the department of Maine-et-Loire. True bugs (Hemiptera: Cimicidae) using bats as hosts have not been well studied in France. Studies on bat guano deposits could provide data on the species distribution. For instance, this method provided new records of *Cimex* sp. and *Cimex dissimilis* (Horváth, 1910) in a roosting colony of *M. myotis* in June 2017 and June 2018 in north-eastern France [177] (about *C. dissimilis* in roosting colony of *M. myotis*, see also [121] and [224]).

##### 1.2.4 Order Siphonaptera Latreille, 1825

Twelve species are known to be associated with bats in France. They belong to the family Ischnopsyllidae Wahlgren, 1907. The species are *Araeopsylla gestroi, Ischnopsyllus (Hexactenopsylla) hexactenus, I. (Ischnopsyllus) elongatus*, *I. (Ischnopsyllus) intermedius, I. (Ischnopsyllus) octactenus, I. (Ischnopsyllus) simplex, I. (Ischnopsyllus) variabilis, Nycteridopsylla ancyluris ancyluris*, *N. eusarca, N. longiceps, N. pentactena*, and *Rhinolophopsylla unipectinata unipectinata.* According to Beaucournu and Launay [37], the published records of *Nycteridopsylla dictena* are dubious [146, 227]. Bat fleas have been observed on 20 hosts in France [3, 14, 23, 29, 33, 36–38, 42–44, 46, 85, 86, 90, 95, 141, 142, 152, 156, 157, 167, 177, 200, 204, 219, 227, 228, 231, 242, 257]. These hosts are *E. serotinus, M. schreibersii*, *M. blythii, M. capaccinii, M. daubentonii*, *M. emarginatus*, *M. myotis*, *M. mystacinus*, *M. nattereri*, *Myotis* sp., *N. leisleri*, *N. noctula*, *P. kuhlii, P. nathusii*, *P. pipistrellus*, *P. auritus*, *P. austriacus*, *R. euryale, R. ferrumequinum*, *R. hipposideros*, *Rhinolophus* sp*.,* and *Tadarida teniotis.* Studies on bat fleas have a long history in France and the book of Walckenaer on insects, entitled *Faune parisienne, insectes ou Histoire abrégée des insectes des environs de Paris* [257], may be among the earliest such works. According to the Inventaire National du Patrimoine Naturel, 91 autochthonous (indigenous) species of the Siphonaptera order have been reported in France. Species of the Ischnopsyllidae found in France represent almost 13% of fleas in the country [153].

### 2 Phylum Nematoda Diesing, 1861

In France, the helminth fauna of bats is varied, with more than 30 species. Nematodes recorded in bats in France are divided into three orders: Muspiceida Bain & Chabaud, 1959, Rhabditida Chitwood, 1933, and Strongylida Molin, 1861; and six families: Onchocercidae Leiper, 1911 (three species), Molineidae Skrjabin & Schulz, 1937 (four species), Rictulariidae Railliet, 1916 (one species), Muspiceidae Bain & Chabaud, 1959 (two species), Seuratidae Hall, 1916 (one species), and Strongylacanthidae (Yorke & Maplestone, 1926, subfamily) Chabaud, 1960 (one species). Thirteen species and two nematodes identified to genus-level have been reported: *Litomosa dogieli*, *L. filaria*, *L. ottavianii*, *Molinostrongylus alatus*, *M. ornatus*, *M. panousei*, *M. tipula*, *Pterygodermatites (Neopaucipectines) bovieri*, *Riouxgolvania nyctali*, *R. rhinolophi*, *Seuratum mucronatum, Strongylacantha glycirrhyza*, *Rictularia* sp., *Riouxgolvania* sp., and *Trichosomum speciosum.* These parasites were documented in the following bat species: *M. schreibersii*, *M. blythii, M. emarginatus*, *M. myotis*, *Plecotus* sp., *Rhinolophus* sp. and three species of the genus *Rhinolophus* (*R. euryale, R. ferrumequinum*, and *R. hipposideros*) [19–21, 54, 56, 57, 68, 75, 86, 97, 99, 167, 177, 194, 200, 211, 233, 235, 243, 245]. The original description of the rare nematode *P. (Neopaucipectines) bovieri* is based on material from *M. myotis* in France. To my knowledge, it is the first published observation in the country (dated September 1885) [56, 66, 230]. The original descriptions of *Riouxgolvania nyctali* and *R. rhinolophi* are based on material from *M. blythii* and *R. euryale* in the Netherlands and the French departments of Ariège and Pyrénées-Orientales [20, 21]. A re-description of *Seuratum mucronatum* was based on material from *Plecotus auritus* (dated 1950) in the French department of Indre-et-Loire [54]. As a comparison, according to Horvat *et al.* [144, 145], two Nematode species (associated with *M. myotis* and *R. ferrumequinum*) are known in Serbia to be associated with bats, whilst in Croatia, these authors noted three species.

### 3 Phylum Platyhelminthes Minot, 1876

#### 3.1 Class Trematoda

Records of 15 recognised species of trematodes from bats have been found from over 17 published papers. They belong to the order Plagiorchiida La Rue, 1957 and are divided into three families: Lecithodendriidae Lühe, 1901 (13 species), Mesotretidae Poche, 1926 (one species), and Plagiorchiidae Lühe, 1901 (one species). These species are *Allassogonoporus amphoraeformis*, *Lecithodendrium granulosum*, *L. linstowi*, *L. moedlingeri*, *Mesotretes peregrinus, Parabascus duboisi*, *P. lepidotus*, *P. semisquamosus, Plagiorchis vespertilionis*, *Prosthodendrium (Prosthodendrium) carolinum*, *P. (Prosthodendrium) hurkovaae*, *P. (Prosthodendrium) chilostomum*, *P. (Prosthodendrium) longiforme*, *P. parvouterus*, and *Pycnoporus heteroporus* [1, 57, 60, 61, 68, 71, 86–89, 97, 163, 167, 177, 188, 200, 202, 243]. Two trematodes identified to genus-level have been reported (*Lecithodendrium* sp. and *Prosthodendrium* sp.) [86]. These parasites were documented in the following bat species: *Eptesicus serotinus* [68, 88, 167], *Miniopterus schreibersii* [68, 167, 188, 200, 235], *Myotis daubentonii* [89, 167], *M. capaccinii* [68, 167], *M. emarginatus* [68, 86, 167], *M. myotis* [57, 61, 68, 167], *Pipistrellus kuhlii* [86], *P. pipistrellus* [57, 60, 61, 68, 86, 97, 163, 167, 235], *Plecotus auritus* [86, 167], *P. austriacus* [68, 167], *Rhinolophus euryale* [68, 167], *R. ferrumequinum* [68, 86–88, 167, 188, 200, 243], and *R. hipposideros* [68, 86, 167, 188, 200, 235].

As a comparison, in the United Kingdom, Lord *et al.* [181, 182] noted four trematode species and one trematode identified to genus-level in bats. In Serbia, Horvat *et al.* [144, 145] noted a total of seven trematode taxa associated with bats, and only one species in Croatia. According to the Inventaire National du Patrimoine Naturel, 28 species of the family Lecithodendriidae and 27 species of the family Plagiorchiidae have been reported in France. Species of the Lecithodendriidae found in bats represent almost 46% of the family [153]. The observations on *Mesotretes peregrinus* (Braun, 1900), published by Combes & Clerc, Dubois and Matskási [68, 88, 188], are of particular interest since this is the only species of the family Mesotretidae reported in Europe [125, 153]. As regards *Plagiorchis vespertilionis* (O.F. Müller, 1784) [68, 86–89, 200, 235], it is worth noting that this species is the only member of the family Plagiorchiidae reported from bats in France. According to Lanza [167], 18 taxa of the Plagiorchiidae family have been reported worldwide. The *Histoire naturelle des helminthes ou vers intestinaux* (1845) by Félix Dujardin (1801–1860) appears to be the earliest French source mentioning bat-associated trematodes [93]. The first documented reports from France, *P. (Prosthodendrium) chilostomum* and *P. heteroporus*, were published in the *Notices helminthologiques* (deuxième série) by Raphaël Blanchard (1857–1919) [57].

#### 3.2 Class Cestoda

Three recognised and one innominate species of cestodes have been reported in bats in France, which makes it the richest community reported in Europe. These species are *Milina grisea*, *Vampirolepis acuta*, and *V. balsacii* [68, 86, 158, 159, 167, 177, 180, 196, 200, 243, 255, 262]. They belong to one family: Hymenolepididae Ariola, 1899 (Cyclophyllidea van Beneden *in* Braun, 1900 order). In addition to these species, one cestode identified to genus-level has been reported (*Vampirolepis* sp.) [86]. The description of *H. balsacii* is based on material from *Myotis bechsteinii* and *Eptesicus serotinus* from north-eastern France collected by the naturalist Henri Heim de Balsac (1899–1979) at a place called Buré d’Orval in Allondrelle-la-Malmaison [158, 159]. Some authors have noted that the observation was made at “Buré” ([158] see also [142]). Joyeux & Baer’s paper on cestodes, entitled *Sur quelques Cestodes de France*, is the first work on the bat cestodes in France (published in 1934 in the journal *Archives du Muséum national d’Histoire naturelle*). Bat cestodes are not well studied, especially in France, where the most recent data from field research is almost 50 years old [68, 86, 158, 159, 167, 177, 180, 196, 200, 243, 255, 262]. France has the most diverse community reported in the literature. According to Frank *et al.* [121], three species are known to be associated with bats in Poland and Hungary. These authors also reported one species in Germany and two species in Austria. It is worth noting that, according to the Inventaire National du Patrimoine Naturel, 75 species of the family Hymenolepididae have been reported in France and species of the family found in bats represent 4% of the family [153].

### 4 Hosts and geographical distribution of bat-parasite associations

**Figure 4:**
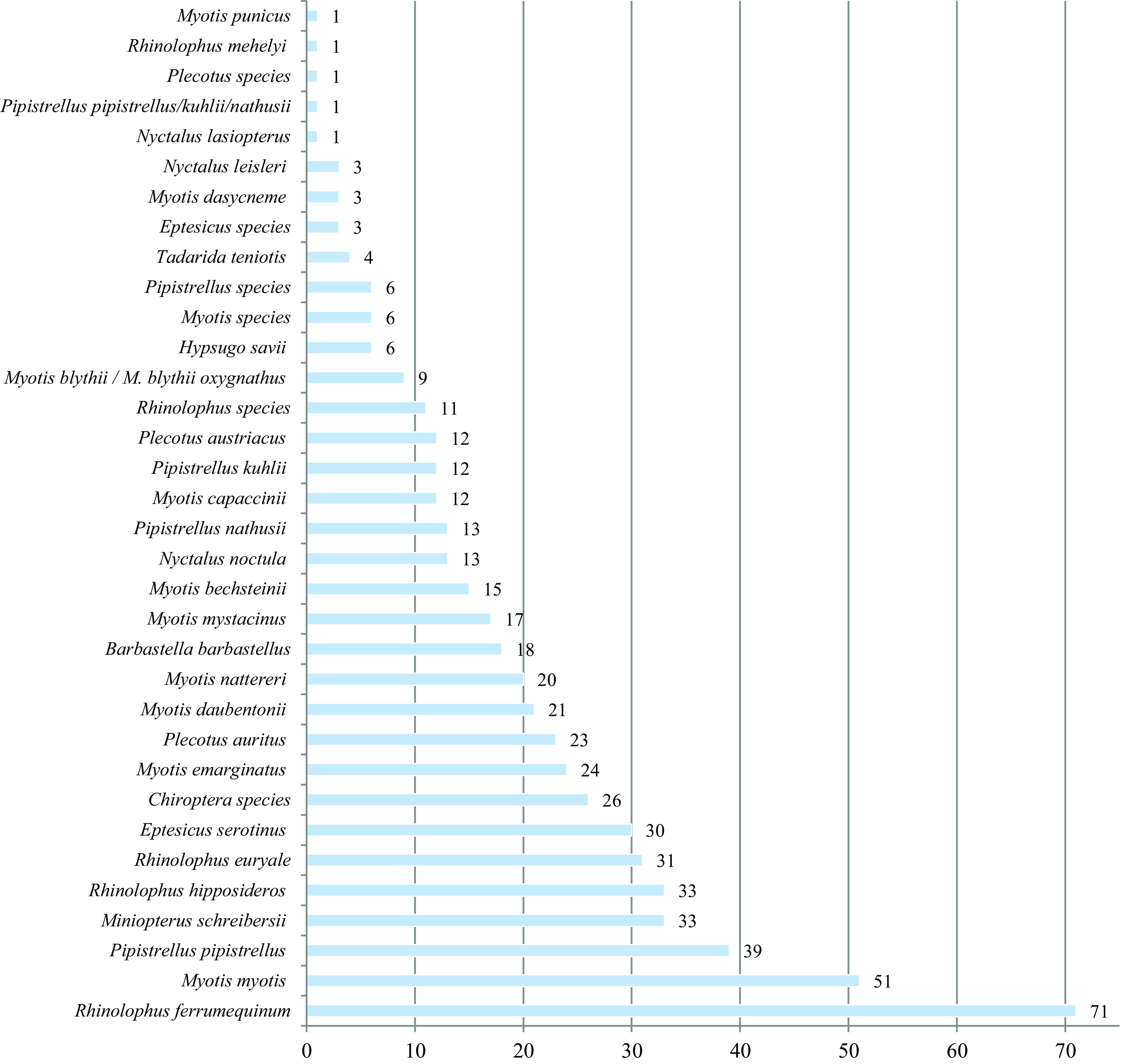
Histogram showing the number of studies (*n* = 237) per host taxon (*n* = 34; species: 27; complex: 1; genera: 6) during the period 1762–2018.

Over a 256-year period, the 113 recognised taxa of bat parasites from France were collected from 27 bats species and six other bats that were not identified to species-level (five genera and the *Pipistrellus* species complex) (Figs. 2 and 4). The taxa are *B. barbastellus, E. serotinus, H. savii, M. schreibersii, M. bechsteinii, M. blythii, M. capaccinii, M. dasycneme, M. daubentonii, M. emarginatus, M. myotis, M. mystacinus, M. nattereri, M. punicus, N. lasiopterus, N. leisleri, N. noctula, P. kuhlii, P. nathusii, P. pipistrellus, P. auritus, P. austriacus, R. euryale, R. ferrumequinum, R. hipposideros, R. mehelyi, T. teniotis, Eptesicus* sp*., Myotis* sp., *Pipistrellus* sp., *Plecotus* sp., *Rhinolophus* sp*.,* and the species complex *Pipistrellus pipistrellus/kuhlii/nathusii*. These species represent almost 79% of the bat fauna of France (including Corsica). The most commonly reported hosts, which are mentioned in more than 29 papers, are *E. serotinus, R. euryale, R. hipposideros, M. schreibersii, P. pipistrellus, M. myotis*, and *R. ferrumequinum.* However, the most cited species is the Greater horseshoe bat (*R. ferrumequinum*); 30% of the analysed publications deal with this species. Some bat species have no records of associated metazoan parasites in the analysed publications (*n* = 237), because the ecology of the host is poorly studied (*E. nilssonii, Vespertilio murinus* (Particoloured Bat)*, M. alcathoe, M. escalerai* and *P. macrobullaris*). In addition to this, since *P. pygmaeus* was identified in the 1990’s, we cannot rule out that some of the records of *Pipistrellus* sp. or *P. pipistrellus* may refer to *P. pygmaeus* [41, 45, 53, 58, 142].

**Figure 5:**
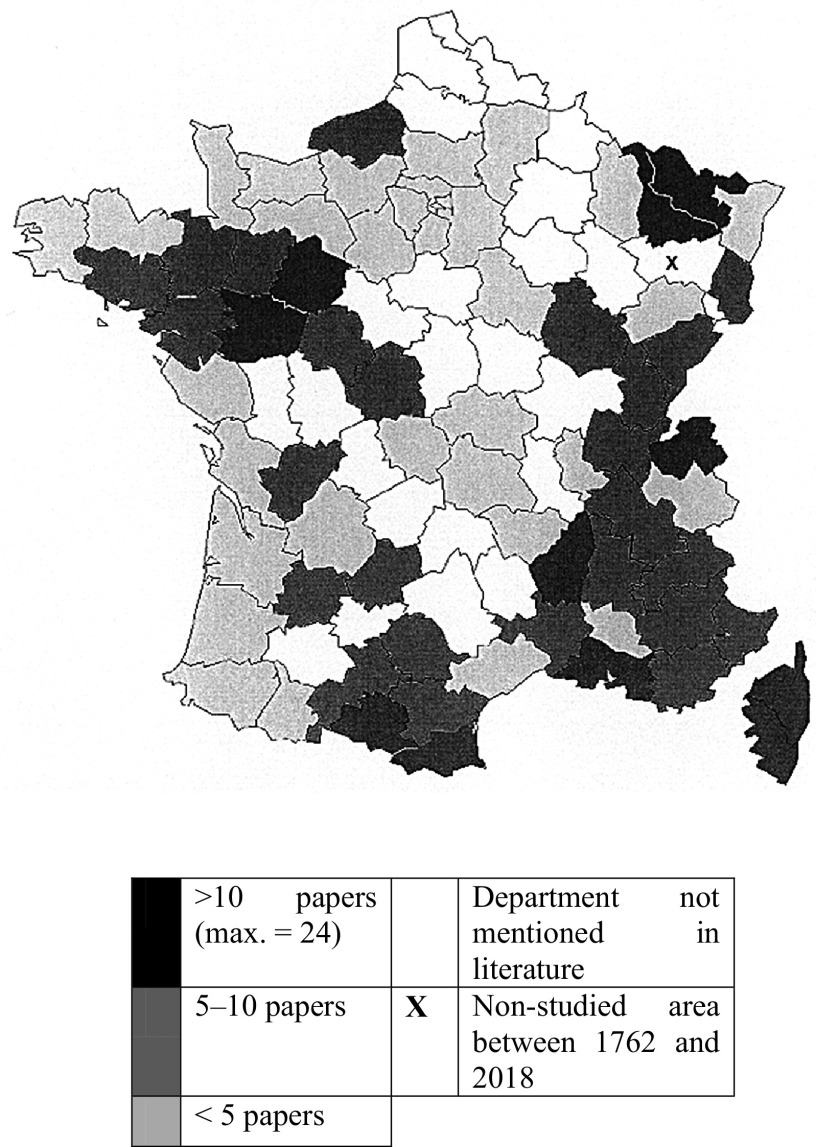
Study area and the number of publications that include data on parasites of bats in each French administrative region (department).

Published field data originated from 72 French departments (Fig. 5). One of them is mentioned as a non-prospected area (department of Vosges). Indeed, there is no publication about bat parasites in this department. Associations with specified geographical locations were most commonly from Ardèche (11 papers), Ariège (13 papers), Bouches-du-Rhône (15 papers), Haute-Savoie (12 papers), Maine-et-Loire (19 papers), Moselle (11 papers), Meurthe-et-Moselle (23 papers), Pyrénées-Orientales (24 papers), Sarthe (12 papers), Haute-Corse, and Corse-du-Sud (23 papers). Importantly, these distribution patterns are influenced more by biased sampling efforts than by actual geographical and ecological patterns. This distribution map only helps to point to well-studied areas. The relative prominence of the departments Ardèche, Ariège, Pyrénées-Orientales, Haute-Corse and Corse-du-Sud compared to other departments is most likely a result of the attention given to karstic areas by European biospeleology (for instance [14, 23–25, 48–53, 69, 70, 115, 118, 120, 126, 131–133, 136, 137, 151, 152, 154, 178, 186, 214–216, 246]). This is closely linked to the success and prevalence of *M. schreibersii, R. euryale*, and *R. ferrumequinum* compared to other bat species. This prevalence could be the result of their ability to roost in the summer in limestone areas and underground sites. The focus on *E. serotinus, R. ferrumequinum, R. hipposideros, P. pipistrellus*, and *M. myotis* is the result of their ability to exploit anthropogenic environments (i.e. farmland, urban areas). As a consequence of this, these species have more contact with human populations (about *P. pipistrellus*, see [182]) and were the first to be studied in France (for the 1762–1844 period see [11, 13, 18, 90–92, 94–97, 124, 183, 220, 258, 260]).

## Conflict of interest

The author declares no conflict of interest.
